# Evaluation of *Leptospira interrogans* knockdown mutants for LipL32, LipL41, LipL21, and OmpL1 proteins

**DOI:** 10.3389/fmicb.2023.1199660

**Published:** 2023-06-23

**Authors:** Luis G. V. Fernandes, Aline F. Teixeira, Ana L. T. O. Nascimento

**Affiliations:** ^1^Laboratório de Desenvolvimento de Vacinas, Instituto Butantan, São Paulo, SP, Brazil; ^2^Programa de Pos-Graduacao Interunidades em Biotecnologia, Instituto de Ciencias Biomedicas, São Paulo, Brazil

**Keywords:** CRISPR interference, knockdown mutants, virulence, host-pathogen interactions, LipL32, LipL41, LipL21, OmpL1

## Abstract

**Introduction:**

Leptospirosis is a worldwide zoonosis caused by pathogenic and virulent species of the genus *Leptospira*, whose pathophysiology and virulence factors remain widely unexplored. Recently, the application of CRISPR interference (CRISPRi) has allowed the specific and rapid gene silencing of major leptospiral proteins, favoring the elucidation of their role in bacterial basic biology, host-pathogen interaction and virulence. Episomally expressed dead Cas9 from the *Streptococcus pyogenes* CRISPR/Cas system (dCas9) and single-guide RNA recognize and block transcription of the target gene by base pairing, dictated by the sequence contained in the 5′ 20-nt sequence of the sgRNA.

**Methods:**

In this work, we tailored plasmids for silencing the major proteins of *L. interrogans* serovar Copenhageni strain Fiocruz L1-130, namely LipL32, LipL41, LipL21 and OmpL1. Double- and triple-gene silencing by in tandem sgRNA cassettes were also achieved, despite plasmid instability.

**Results:**

OmpL1 silencing resulted in a lethal phenotype, in both *L. interrogans* and saprophyte *L. biflexa*, suggesting its essential role in leptospiral biology. Mutants were confirmed and evaluated regarding interaction with host molecules, including extracellular matrix (ECM) and plasma components, and despite the dominant abundance of the studied proteins in the leptospiral membrane, protein silencing mostly resulted in unaltered interactions, either because they intrinsically display low affinity to the molecules assayed or by a compensation mechanism, where other proteins could be upregulated to fill the niche left by protein silencing, a feature previously described for the LipL32 mutant. Evaluation of the mutants in the hamster model confirms the augmented virulence of the LipL32 mutant, as hinted previously. The essential role of LipL21 in acute disease was demonstrated, since the LipL21 knockdown mutants were avirulent in the animal model, and even though mutants could still colonize the kidneys, they were found in markedly lower numbers in the animals' liver. Taking advantage of higher bacterial burden in LipL32 mutant-infected organs, protein silencing was demonstrated *in vivo* directly in leptospires present in organ homogenates.

**Discussion:**

CRISPRi is now a well-established, attractive genetic tool that can be applied for exploring leptospiral virulence factors, leading to the rational for designing more effective subunit or even chimeric recombinant vaccines.

## Introduction

Leptospirosis is a neglected zoonosis of global concern caused by pathogenic species of the genus *Leptospira*, which can infect a broad range of hosts directly after contact with the urine of infected animals or indirectly after exposure to contaminated water or soil (Bharti et al., 2003; Haake and Levett, 2015). Symptom presentation may vary, ranging from non-specific flu-like symptoms, such as fever, chills, headache, and myalgia, to severe leptospirosis, encompassing a potentially fatal condition known as Weil's disease (Levett, 2001) or severe pulmonary hemorrhage leptospirosis (SPHL) (McBride et al., 2005; Segura et al., 2005; Dolhnikoff et al., 2007), the latter recognized as an emerging clinical manifestation (Nally et al., 2004). Leptospirosis is responsible for more than 1 million cases and almost 60,000 human deaths per year worldwide (Costa et al., 2015; Torgerson et al., 2015).

The molecular basis of leptospiral pathogenesis, despite recent advances in functional genomics and mutagenesis, is still poorly understood (Murray, 2015; Daroz et al., 2021). After contact with damaged skin or mucosa, pathogenic leptospires can rapidly penetrate and breach host biological barriers and migrate across viscous environments (Picardeau, 2017), such as the extracellular matrix (ECM). Many leptospiral adhesins are capable of mediating bacterial adhesion to different elements on the surface of host cells and ECM (Fernandes et al., 2016b; Daroz et al., 2021).

When reaching the bloodstream for dissemination, leptospires need to overcome host natural defenses, such as the complement system and oxidative stress, and several research groups have elucidated the role of surface-exposed proteins in leptospiral immune evasion mechanisms by *in vitro* assays with recombinant proteins (Verma et al., 2006; Vieira et al., 2010a, 2018; Castiblanco-Valencia et al., 2012, 2016; Siqueira et al., 2016; Cavenague et al., 2019). Rapid tissue dissemination can also be related to the ability of pathogenic leptospires to impair the host coagulation cascade (Oliveira et al., 2013; Fernandes et al., 2015, 2016a).

Nevertheless, *in vitro* features of purified recombinant protein do not necessarily reflect the native counterpart role in *Leptospira* spp., and mutations in genes of interest are required to evaluate the resultant phenotype for function confirmation (Shapiro et al., 2018). Accordingly, *in vitro* evidence that leptospiral immunoglobulin-like (Lig) proteins LigA and LigB could play a pivotal role in complement evasion (Castiblanco-Valencia et al., 2012, 2016; Haake and Matsunaga, 2020) was further confirmed by evaluation of a double knockdown LigAB mutant obtained by CRISPR interference (CRISPRi) (Fernandes et al., 2021b), and this mutant was later shown to be avirulent in an animal model (Fernandes et al., 2021c).

The application of CRISPRi for leptospiral mutant generation has allowed the confirmation of native protein function in host–pathogen interaction and virulence. This novel genetic tool for gene silencing relies on the episomal expression of two components: a programmable DNA-binding protein, which is catalytically inactive Cas9 (dCas9), and a single-guide RNA, which dictates the target for gene silencing by base pairing (Qi et al., 2013; Fernandes et al., 2019).

It was previously shown that the silencing of the major outer membrane protein (OMP) LipL32 results in substantial changes in amounts of other lipoproteins and potential virulence factors expressed by *Leptospira*, including the virulence factors LigA and LigB. It was hypothesized that this global proteomic change occurred to fill a niche left by the silencing of LipL32 (Fernandes et al., 2021c). Moreover, the virulence evaluation of this mutant hinted at augmented virulence.

Outer membrane proteins (OMPs) are considered promising candidates for virulence factors because of their location, which enables direct interaction with host components and cells (Haake and Matsunaga, 2002; Ko et al., 2009). In leptospires, lipoproteins are the predominant OMPs, similar to *Borrelia* spp. (Haake and Matsunaga, 2010). Quantitative proteome analysis conducted by Malmström et al. (2009) revealed that LipL32, Loa22, LipL41, and LipL21 are the most abundant lipoproteins in *L. interrogans*. Notably, Loa22 was the first genetically defined virulence factor identified in *L. interrogans* (Ristow et al., 2007).

In this context, it is possible that other major proteins could also play a role in the virulence of leptospires. Exploring and validating leptospiral virulence factors could ultimately favor the design of more effective subunit or even chimeric recombinant vaccines (Barazzone et al., 2021). Directing the immune responses toward virulence determinants has historically proven to be a successful approach in interrupting the cycle of infection, leading to the generation of protective immunity (Delany et al., 2013).

LipL41, an abundant lipoprotein of pathogenic leptospires, is a 41 kDa protein first identified by Haake et al. (1991) and later described by Shang et al. (1996). Furthermore, LipL41 was previously reported to elicit a synergistic protection profile along with OmpL1 protein against lethal challenge in hamsters, inferring that OmpL1 protein could be relevant to pathogenesis (Haake et al., 1999). It was shown that LipL21 can contribute to leptospiral virulence as its binding to peptidoglycan allows leptospires to escape NOD1 and NOD2 (Ratet et al., 2017). In addition, LipL21 was shown to be a potent myeloperoxidase inhibitor, an enzyme stored in neutrophils (Vieira et al., 2018). A transposon mutant of *L. interrogans* serovar Manilae lacking LipL21 was found to be avirulent in a hamster model. However, complementation failed to restore the mutant's virulence. Thus, the contribution of LipL21 to the virulence of leptospires remains unclear (Ratet et al., 2017).

Hence, we were interested in evaluating the outcomes of the silencing of genes coding for these major proteins. For that, we used CRISPRi to create mutants of the major proteins LipL32, LipL41, LipL21, and OmpL1 in *L. interrogans* serovar Copenhageni, and mutant bacteria were evaluated regarding their interaction with host molecules and virulence in a hamster model.

## Materials and methods

### Bacterial strains and plasmids

Virulent low-passage *L. interrogans* serovar Copenhageni strain Fiocruz L1-130 and saprophytic *L. biflexa* serovar Patoc strain Patoc1 were cultured in EMJH medium (Difco, BD, Franklin Lakes, NJ, United States) supplemented with 10% (vol/vol) *Leptospira* Enrichment EMJH (Difco) (Turner, 1970) and 1% rabbit serum. Solid media were prepared by supplementing with 1.2% Noble agar (Difco). For transconjugant recovery, spectinomycin was added at a concentration of 40 μg/mL. *E. coli* strain β2163 auxotrophic for diaminopimelic acid (DAP) (Demarre et al., 2005; Picardeau, 2008) was used for general cloning and as conjugation donor cells and was grown in Luria–Bertani (LB, Difco) medium supplemented with DAP (0.3 mM, Sigma).

### Macromolecules and antibodies

Murine laminin (Engelbreth-Holm-Swarm murine sarcoma basement membrane, L2020), collagen type IV (Engelbreth-Holm-Swarm murine sarcoma basement membrane, C0543), human cellular (foreskin fibroblasts, F2518) and plasma fibronectin (F2006), human fibrinogen (F4883), plasminogen (PLG) (P7999), recombinant E-cadherin (5085), thrombin (605190), and bovine serum albumin (BSA; A3912) were acquired from Sigma-Aldrich. Anti-laminin (L9393), anti-fibronectin (F3648), anti-fibrinogen (F8512), anti-collagen (SAB5500022), and anti-E-cadherin (SAB5500022) antibodies were acquired from Sigma-Aldrich, and anti-PLG antibody was produced *in house*. In brief, BALB/c female mice were immunized subcutaneously with 10 μg of human PLG mixed with 10% (v/v) Alhydrogel [2% Al(OH)3; Brenntag Biosector] as an adjuvant. Two subsequent boost immunizations were given at 2-week intervals. Horseradish peroxidase (HRP)-conjugated secondary anti-mouse (A9044), anti-goat (A8919), and anti-rabbit IgG (A0545) antibodies were also purchased from Sigma.

### Plasmid construction and *Leptospira* spp. conjugation

Plasmid pMaOri.dCas9 (Fernandes et al., 2019) was used as a backbone for sgRNA cassette ligation by Gibson assembly (New England BioLabs, E2611S), according to manufacturer's instructions. The plasmid was digested at the *Xma*I site and sgRNA cassette targeting *lipL32* was amplified from previously described pMaOri.dCas9sgRNAlipL32 (Fernandes et al., 2021b), with primers sgRNA F and sgRNA R (Table 1) designed for the Gibson assembly reaction and for the specific reconstruction of the *Xma*I site at only the 5′ end of the inserted cassette. Thus, this newly obtained pMaOri.dCas9sgRNAlipL32 in this study differs from a previous one (Fernandes et al., 2021b), which was obtained by T4 ligase-mediated reaction and displayed *Xma*I sites at both ends of the cassette.

**Table 1:** Primer sequences used in this study.

**Primer**	**Sequence (5^′^ → 3^′^)**	**Purpose**
sgLipL21 F	AACGTCTTTCGGATCGGATCGTTTTAGAGCTAGAAATAGC	Substitute the *lipL32* protospacer in the sgRNA cassette for the *lipL21* protospacer
sgLipL21 R	GATCCGATCCGAAAGACGTTGAAAATCACGGTATGAACTTAGG	
sgLipL41 F	CTACGTTACGAATGGTTCCGGTTTTAGAGCTAGAAATAGCAAG	Substitute the *lipL32* protospacer in the sgRNA cassette for the *lipL41* protospacer
sgLipL41 R	CGGAACCATTCGTAACGTAGGAAAATCACGGTATGAACTTAGG	
sgOmpL1 F	GCCGCCAGTAGTTCTATCGAGTTTTAGAGCTAGAAATAGCAAG	Substitute the *lipL32* protospacer in the sgRNA cassette for *ompL1* protospacer
sgOmpL1 R	TCGATAGAACTACTGGCGGCGAAAATCACGGTATGAACTTAGG	
sgOmpL1biflexa F	CGAAACGTACGTTTTCTTTGGTTTTAGAGCTAGAAATAGC	Substitute the *lipL32* protospacer in the sgRNA cassette for the *L. biflexa ompL1* protospacer
sgOmpL1biflexa R	CAAAGAAAACGTACGTTTCGGAAAATCACGGTATGAACTTAGG	
sgRNA F	TTAGGATCCCCCGGGGAACAAGAAAGAGTCAGAG	Amplification of whole sgRNA cassette for Gibson assembly
sgRNA R	ATCGAATTCCTGCAGAAAAAAGCACCGACTCGGTGC	
sgRNAsecond R	CTTTCTTGTTCCCGGAAAAAAGCACCGACTCGGTGC	
pMaOri2 F	ACGCAATGTATCGATACCGAC	Amplification flanking the sgRNA cassette
pMaOri2 R	ATAGGTGAAGTAGGCCCACCC	
qLipL32 F	AAGGATCTTTCGTTGCATCT	Quantification of bacterial load
qLipL32 R	TTACTTAGTCGCGTCAGAAG	

To generate plasmids containing a single sgRNA cassette targeting either *lipL41, lipL21*, or *ompL1* genes, we followed the protocol previously described by Fernandes and Nascimento (2020) to define protospacers (Table 2). The existing protospacer sequence in the template pMaOri.dCas9sgRNAlipL32 plasmid was substituted with the desired protospacer sequence by PCR. Specifically, primers flanking the 20-nt protospacer were used and included the new desired protospacer sequence at the 5' end to provide the required overhang. This resulted in a 9.5 kb amplicon, which was purified and used for subsequent Gibson assembly reactions to seal the plasmid backbone.

**Table 2:** Target genes and protospacers.

**Target gene**	**Protospacer (5^′^ → 3^′^)**	**Species**
*lipL32*	ACCACCGAAAGCACCACAAG	*L. interrogans*
*lipL41*	CTACGTTACGAATGGTTCCG	*L. interrogans*
*lipL21*	AACGTCTTTCGGATCGGATC	*L. interrogans*
*ompL1*	GCCGCCAGTAGTTCTATCGA	*L. interrogans*
*ompL1*	CGAAACGTACGTTTTCTTTG	*L. biflexa*

To create the double sRNA cassette plasmids, the pMaOri.dCas9sgLipL41 was digested with *Xma*I enzyme, and the desired cassettes for *lipL32* or *ompL1* were amplified from their respective single sgRNA plasmids with primers sgRNA F and sgRNA second R (Table 1). Plasmid and inserts were mixed at 1:5 proportion for Gibson assembly. All the reactions were used to transform *E. coli* β2163 cells, which were further seeded onto LB plates containing DAP (0.3 mM) and spectinomycin (40 μg/mL) and individual colonies selected by PCR with pMaOri2 F and R primers flanking the sgRNA cassettes (Table 1). For the triple sgRNA plasmids, pMaOri.dCas9sgRNAlipL32sgRNAlipL41 was digested with *XmaI* enzyme, and the cassette for *lipL21* was obtained and ligated as described above. All the obtained plasmids were sequenced with pMaOri2 primers to confirm the correct sgRNA cassettes.

*Leptospira* spp. knockdown mutants were obtained by conjugation as previously described (Fernandes et al., 2021a).

### Evaluation of mutants' binding to host ECM and plasma components

Before binding experiments, each component was serially diluted (1–0.008 μg in 100 μL) and coated onto ELISA plate wells (Costar High Binding; Corning) for detection with appropriate anti-component and secondary antibodies. The immobilized mass that gave a linear curve beyond that point was selected for incubation with the leptospiral mutant strains.

Knockdown mutants or control cells containing only pMaOri.dCas9 were brought to the same concentration (5 × 10^8^/mL) and incubated with the previously defined components' mass for 2 h at 30°C; the bacterial suspensions were then centrifuged (10,000 × g, 15 min), and the supernatant was carefully removed and submitted to another round of centrifugation and supernatant collection. Next, 100 μL of each solution were used to coat the wells for 16 h at room temperature, in seven replicates, and a control without component was used as a blank to determine the baseline signal of the reaction. In parallel, a component mass curve was run in the experiment to determine the percentage of unbound components and, by consequence, indirectly infer the component bound to the leptospiral surface. Plates were washed with PBS containing 0.05% Tween-20 (PBS-T), blocked with 3% BSA in PBS-T for 1 h, and incubated with antibodies for each component (1 h 37°C) and secondary horseradish peroxidase (HRP)-conjugated secondary antibodies in 1% BSA in PBS-T. The wells were washed six times, and o-phenylenediamine (1 mg/mL) in citrate phosphate buffer (pH 5.0) plus 1 μL/mL H_2_O_2_ was added (100 μL per well).

The reaction proceeded for 10 min and was interrupted by the addition of 50 μL of 2 M H_2_SO_4_. Readings were taken at 492 nm with a microplate reader (Multiskan EX; Thermo Fisher Scientific, Helsinki, Finland), and absorbance values were compared with values generated from the component curve. The residual component mass in the supernatant was used to infer the mass bound to the leptospiral surface, statistical analysis was performed using one-way ANOVA, followed by the Tukey post-test for pairwise comparisons of each group against control dCas9, and a *P*-value of < 0.05 was considered to be statistically significant.

### Fibrin clot inhibition by mutant strains

Leptospires were recovered by centrifugation (10,000 × g, 15 min), washed twice with 0.9% NaCl, and resuspended in 0.9% NaCl to the standardized OD_420nm_ of 5; next, 60 μL of the leptospiral suspension were mixed with 540 μL of a human fibrinogen solution (1 mg/mL in 0.9% NaCl), resulting in a final leptospiral OD_420nm_ of 0.5. After 2 h incubation at 37°C, 90 μL of the incubation reaction were added to each well of a microdilution plate and mixed with 10 μL of 0.5 U/mL human thrombin. Experiments were performed with six replicates, and a control with no leptospires (maximal fibrin clot formation) or no thrombin (no fibrin clot formation) was included. Plates were read at 600 nm at 2-min intervals.

### Plasmin formation

Plasmin formation was evaluated according to protocols described by Vieira et al. (2009), with slight modification. Leptospires were grown in EMJH medium without rabbit serum to avoid PLG contamination, recovered by centrifugation (10,000 × g, 15 min), washed twice, and resuspended in PBS. Bacterial suspensions were standardized to OD_420nm_ of 0.5 in 500 μL of PBS. Next, 2 μg of PLG were added followed by incubation for 2 h. Cells were pelleted by centrifugation, washed once, and resuspended in 250 μL of PBS, to which 50 ng of urokinase-type PLG activator (uPA) and 250 μL of plasmin (PLA) substrate D-Val-Leu-Lys-p-nitroanilide dihydrochloride (Sigma) were added to a final concentration of 0.4 mM for amidolytic activity determination. For experimental controls, either PLG, uPA, or leptospires were omitted from the reactions. After a 16-h reaction, bacteria were pelleted and the supernatant was collected for absorbance readings at 405 nm in microdilution plates, in quintuplicate.

### Animal ethics statement

All animal experimentation was conducted in accordance with protocols as reviewed and approved by the Animal Care and Use Committee at Instituto Butantan under number 8790290422 and as approved by Institutional guidelines. Four- to six-week-old female hamsters were acclimated to the facility a week prior to the challenge and segregated in boxes of four animals with similar general weight average. The weight of each animal was considered 100% on the day of infection. Hamsters were monitored daily and always had *ad libitum* access to food and water.

### Mutant *Leptospira* challenge

LipL32, LipL41, and LipL21 knockdown mutants were cultured to mid-late log phase in EMJH medium at 29°C. Groups of female Syrian gold hamsters (*Mesocricetus auratus*, group *n* = 8, two boxes of *n* = 4) were inoculated intraperitoneally with 5 × 10^7^ leptospiral mutants at the same *in vitro* passage. Each animal was monitored daily and weighed for clinical signs of acute leptospirosis. Animals were humanely euthanized when weight loss (>10%) and/or additional clinical signs (blood on paws/nose/urogenital tract, lethargy, etc.) were observed (Zuerner et al., 2012) or on the basis of experimental time point. One kidney and one liver lobe were harvested and immediately macerated in 5 mL of EMJH medium plus 5-fluorouracil (5-FU). Suspensions were used at different dilutions to inoculate EMJH medium plus 5-FU, without or with spectinomycin, to confirm mutant identities and intact plasmids. Cultures were monitored daily by dark-field microscopy, and positivity scores were recorded depending on cell densities, with values of 0 (no cells observed), 1 (1–10 leptospires per field), 2 (10–100), and 3 (more than 100 leptospires per field). An additional section of the kidney and lobe of the liver was harvested and frozen at −80°C for bacterial burden analysis.

Once the virulence phenotypes of interest were established, a second animal experiment was conducted with additional and alternative experimental endpoints, with the challenge inoculum comprised of 5 × 10^7^ mutant leptospires. One set of animals of each group (*n* = 4) were humanely euthanized on day 4 for synchronized comparison purposes, and the additional set (*n* = 4) was monitored daily for clinical symptoms.

Endpoints of animals inoculated with knockdown strains were compared to those displayed by animals infected with *L. interrogans* containing empty pMaOri.dCas9 plasmids by the log-rank test, and a *p*-value of < 0.05 was considered to be statistically significant.

### Quantification of bacterial load in target organs

A cross section of the kidney and liver tissue (15–20 mg) was used for DNA isolation using the DNeasy DNA Blood and Tissue isolation kit (Qiagen, MD, USA) following the manufacturer's specifications and using a final elution volume of 200 μL. The concentration curve of leptospiral genomic equivalent (GEq) was prepared with genomic DNA from *L. interrogans*. The bacterial load was quantified by quantitative PCR assay based on the protocol described by Wunder et al. (2021) using an CFX96 Real-Time System (Bio-Rad, Hercules, CA, USA). The *lipL*32 gene was amplified in the samples (5 μL template/well) using primers qLipL32F (5**′**AAGGATCTTTCGTTGCATCT 3**′**) and qLipL32R (5**′** TTACTTAGTCGCGTCAGAAG 3**′**), and amplicon was detected by SYBR Green PCR Master Mix (Applied Biosystems, Foster City, CA, USA). The total volume of PCR reactions was 20 μL containing 400 nM of each primer. The amplification protocol consisted of 10 min at 95°C, followed by 40 cycles of amplification (95°C for 15 s and 60°C for 1 min). A result was considered negative if the Ct was >40. The concentration of leptospires (GEq per gram of tissue) was calculated based on the standard curve equation.

Statistical differences between groups of interest were determined by one-tailed Mann–Whitney non-parametric test, and a *p*-value of < 0.05 was considered to be statistically significant.

### Evaluation of phenotype stability *in vivo* and *in vitro*

For evaluating target protein silencing in leptospires sheltered at the target organs, tissue macerates (5 mL) were transferred to 15-mL conical tubes and centrifuged at 1,150 × g for 10 min, and the supernatant (~3 mL) was carefully recovered and transferred to two 1.5-mL microtubes. After centrifugation (10,000 × g, 15 min), pellets were washed twice with PBS and resuspended in 200 μL. Target protein expression in leptospires cultured out of EMJH medium after inoculation with organ macerates with or without spectinomycin (40 μg/mL) was also assessed. Bacteria were recovered from the medium by centrifugation (10,000 × g, 15 min) and washed twice with PBS, and suspensions were then counted. Protein samples were then processed for SDS-PAGE on 12% acrylamide gels (Bio-Rad) according to the manufacturer's guidelines. Cell lysates were applied to represent the total amount of 5 × 10^6^ cells per lane. For comparing the extent of restored expression of target protein, lysates prepared from the control pMaOri.dCas9-containing leptospires were applied to generate different amounts of cells per lane. The immunoblots were performed to infer the maintenance of gene silencing, as previously described (Fernandes et al., 2021b).

## Results

### Transconjugant colony recovery suggests the essential role of OmpL1 porin in both *L. interrogans* and *L. biflexa*

**Figure 1:**
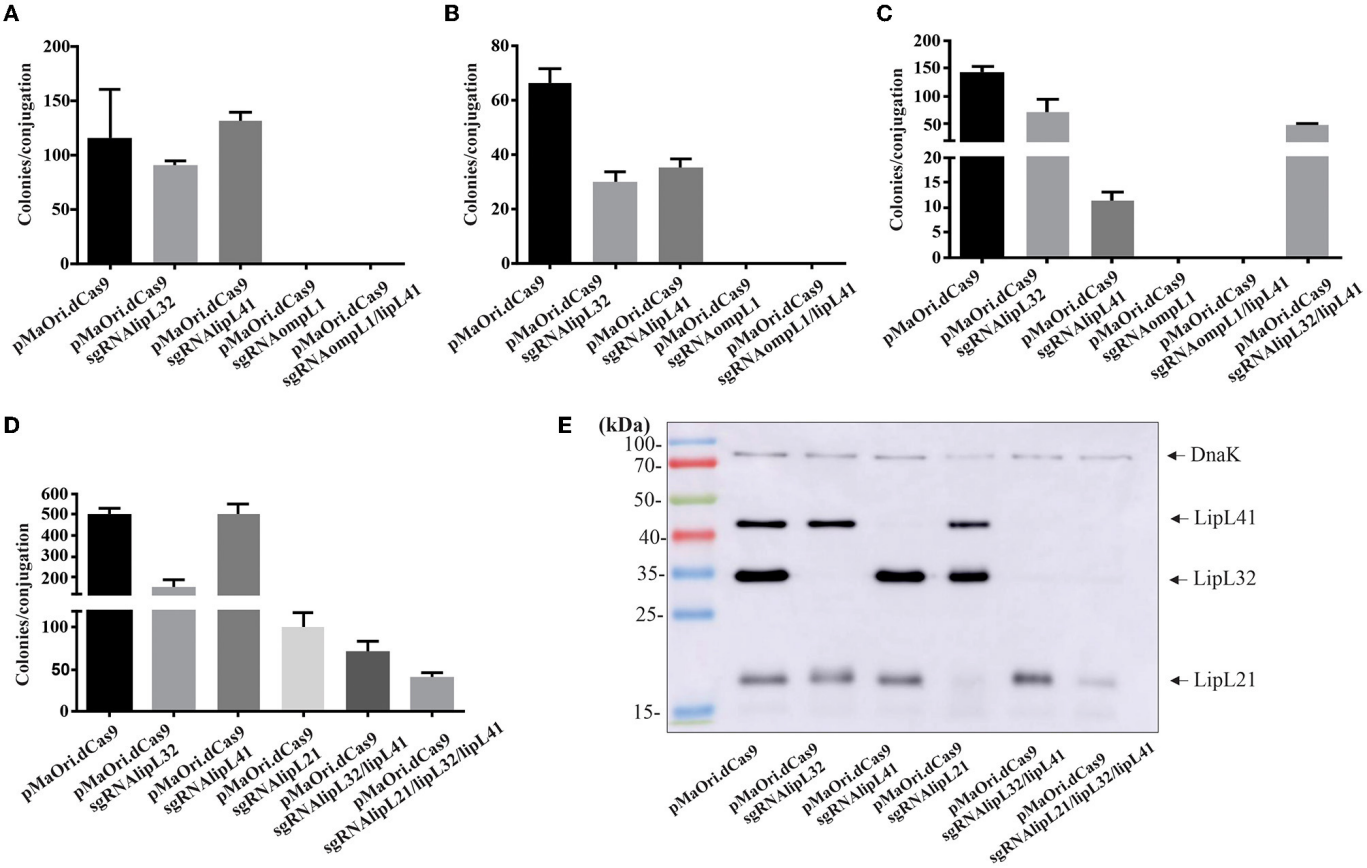
Transconjugant recovery and mutant validation. *E. coli* cells containing the plasmids of interest for gene silencing were used for *Leptospira* conjugation, and colonies formed on EMJH plus spectinomycin plates were counted. Graphics **(A–D)** refer to colony recovery in different experiments. Colonies were recovered from the plates, and leptospiral cells were released from the agar and inoculated in liquid media plus antibiotic. After growth, cells were harvested and whole-cell lysates were evaluated by immunoblotting with anti-LipL32, anti-LipL41, and anti-LipL21 antisera **(E)**. Polyclonal antiserum against DnaK chaperone was used as a loading control.

Initially, aimed at the production of knockdown mutants for *L. interrogans* major membrane proteins, sgRNA cassettes were designed to target the coding strands of the *lipL41* and *ompL1* genes. As previous study suggested a synergetic effect on both proteins in an animal model (Haake et al., 1999), a plasmid containing the abovementioned sgRNA *in tandem* (see Material and Methods) was also constructed for concomitant silencing of LipL41 and OmpL1 proteins. Plasmid pMaOri.dCas9sgRNAlipL32 was also employed as a control for LipL32 silencing. As seen in Figures 1A–C, a markedly high number of transconjugant colonies could be recovered when *L. interrogans* cells carried pMaOri.dCas9 alone or containing the sgRNA for *lipL32* or *lipL41*. Surprisingly, no colonies were observed across the experiments when the plasmid constructions contained the sgRNA targeting *ompL1*, alone or in tandem with sgRNA for *lipL41*, suggesting that OmpL1 porin could be essential for leptospiral basic biology.

Since the double mutant for OmpL1 and LipL41 was unachievable, a plasmid containing sgRNA for *lipL32* and *lipL41* was created for the demonstration of multiple silencing by CRISPRi, and after conjugation, a substantial number of transconjugant colonies were observed (Figures 1C, D). Pushing the boundaries of multiple silencing, cassettes for concomitant *lipL21, lipL32, lipL41*, and *lipL21* alone were used for the conjugation of *E. coli* cells to *L. interrogans* (Figure 1D). This last experiment was performed for the purpose of obtaining synchronized virulent mutants for downstream characterization and evaluation.

Transconjugants recovered were validated by immunoblotting, and gene silencing of the synchronized mutants (Figure 1D) is depicted in Figure 1E. As expected, when *L. interrogans* contained the plasmids for the expression of dCas9 and the sgRNA to *lipL32* and *lipL41* individually, no bands corresponding to the respective proteins were observed in the whole-cell lysates. For LipL21, a drastic reduction in protein expression was observed (>90%) when membranes were overexposed (Figure 1E), most possibly due to the selected protospacer location at the end of the *lipL21* gene. Aimed at demonstrating the applicability of multiple gene silencing by multiple sgRNA cassettes in the same plasmid, recombinant cells containing plasmid for dCas9 and sgRNA for both *lipL32* and *lipL41* or the triple cassette for *lipL21, lipL32*, and *lipL41* (Figure 1E) were also evaluated, indicating that the multiple one-step silencing strategy is suitable for *Leptospira*.

However, evaluation of double mutants after just one passage in a medium with antibiotic indicates an undesirable plasmid instability, which is denoted by the restored expression of the target proteins (Supplementary Figure 1); it is worth mentioning that the same difficulty was observed with the triple mutants. This phenomenon was observed with the mutants retrieved in every experiment and is most probably because of the repetitive elements due to the *in tandem* architecture of the sgRNA cassettes, favoring recombination events (Oliveira et al., 2009). Sequencing of the amplicons obtained after plasmid amplification with pMaOri2 primers in one of the clones for the double mutant indicated that recombination occurred resulting in the loss of the *lipL41* protospacer, which was replaced by another *lipl32* one. In addition, since recombination was confirmed to be happening, a complete loss of the whole sgRNA cassettes in the population cannot be ruled out.

To evaluate whether the *ompL1* gene is essential to *Leptospira* spp., a sgRNA targeting the *ompL1* gene in the saprophyte *L. biflexa* was designed, which shares 44% amino acid identity with the annotated *L. interrogans ompL1* ortholog. Confirming the findings in the pathogen *L. interrogans*, only a few (1st experiment) or no (2nd experiment) saprophyte colonies were retrieved when *ompL1* was silenced (Supplementary Figure 2A), in contrast to tens of thousands of colonies recovered when control pMaOri.dCas9 plasmid was used, indicating the essential role of OmpL1 protein. Possibly, the few colonies observed in the first experiment are spontaneous mutants to spectinomycin, which has been shown to appear in conjugation and transformation assays (Poggi et al., 2010; Fernandes et al., 2019). Representative plates showing the colonies are depicted in Supplementary Figure 2B.

### Effect of gene silencing on interaction with host components

Supernatants after interaction of E-cadherin (10 μg/mL), collagen IV (0.625 μg/mL), laminin (1 μg/mL), plasma (0.05 μg/mL), cellular fibronectin (0.2 μg/mL), and fibrinogen (0.1 μg/mL) with distinct *Leptospira* mutants were used to coat ELISA plates for component detection, along with a concentration curve for determining the percentage of component bound to the leptospiral surface.

**Figure 2:**
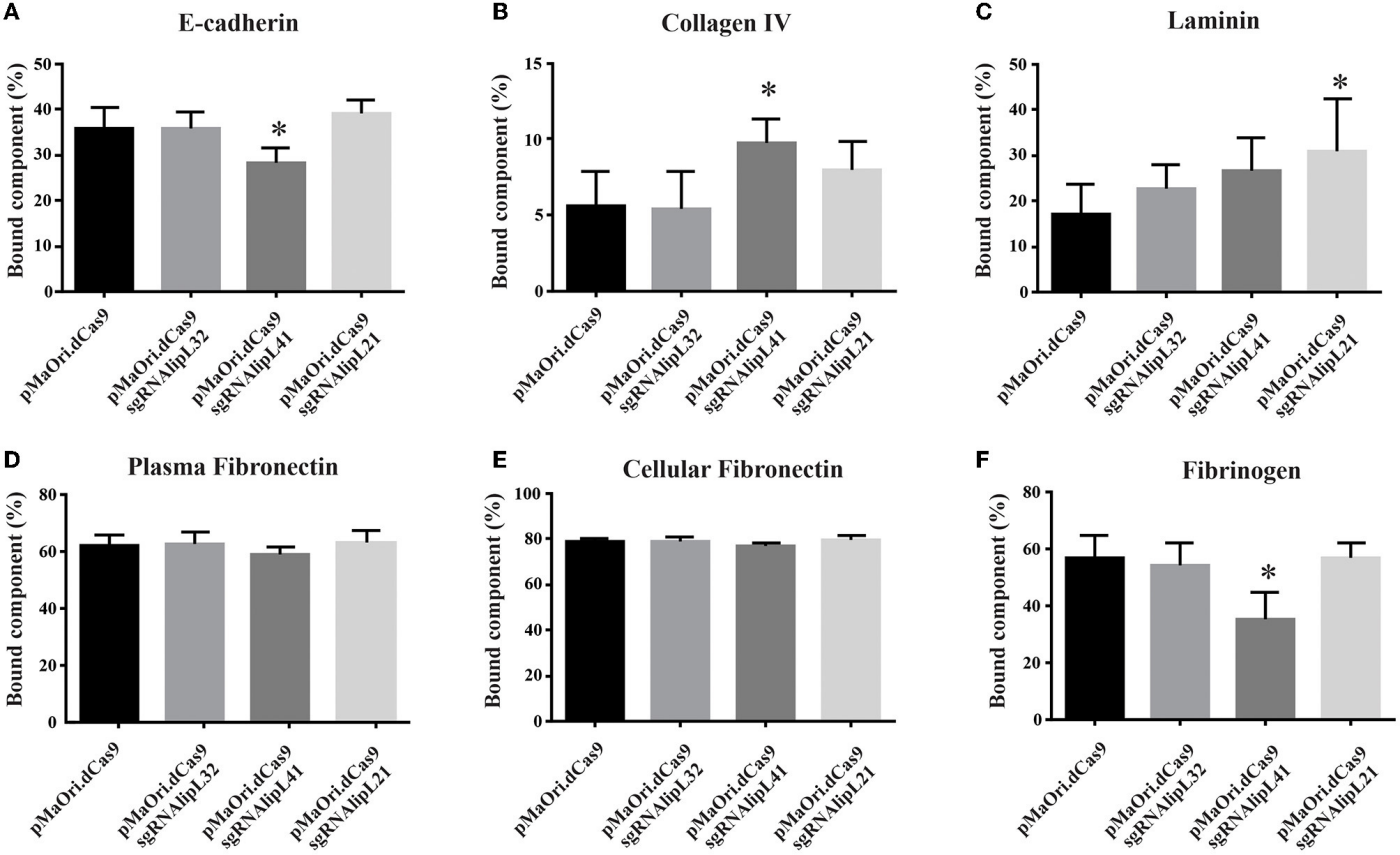
Interaction of *L. interrogans* mutant cells with host components. *L. interrogans* containing empty pMaOri.dCas9 (control) or knockdown mutants to LipL32 (pMaOri.dCas9sgRNAlipL32), LipL41 (sgRNAlipL41), and LipL21 (sgRNAlipL21) were incubated (5 × 10^8^ cells/mL) with optimized concentrations of host components E-cadherin **(A)**, collagen type IV **(B)**, laminin **(C)**, plasma **(D)**, and cellular **(E)** fibronectin and fibrinogen **(F)**. Bacterial suspensions after incubation were centrifuged, and the resulting supernatant was removed and submitted to a second round of centrifugation. The resulting supernatants were used to coat ELISA plates for component detection, along with a concentration curve. Plates were washed, blocked with 3% BSA in PBS-T, incubated with antibodies for each component and horseradish peroxidase (HRP)-conjugated secondary antibodies. Reactivity was revealed with o-phenylenediamine substrate, and the residual component mass in the supernatant was used to infer the mass bound to the leptospiral surface. Statistical analysis was performed using one-way ANOVA, followed by the Tukey post-test for pairwise comparisons of each group against control dCas9, and a *P*-value of < 0.05 (*) was considered to be statistically significant.

In regard to E-cadherin binding, a significant decrease was achieved only for the LipL41 mutant in comparison with the control containing only dCas9 (*P* < 0.05) (Figure 2A), whereas an increase in binding was observed for the same mutant to collagen IV (Figure 2B); this increased binding might be due to an upregulation of leptospiral proteins that display higher affinity for the component. Major proteomic changes were previously demonstrated for the LipL32 knockdown mutant (Fernandes et al., 2021c). Accordingly, binding to laminin was also increased in the LipL21 mutant (*P* < 0.05) in comparison with the dCas9 control (Figure 2C). No differences were observed in binding to either plasma or cellular fibronectin (Figures 2D, E).

Even though binding to fibrinogen was affected when LipL41 protein was silenced (*P* < 0.05) (Figure 2F), no differential effect on fibrin clot inhibition was observed after incubation of fibrinogen with the mutants compared to the control cells containing only pMaOri.dCas9 plasmid (Supplementary Figure 3).

Overall, despite the statistical significances obtained, no substantial changes were observed in the differential interaction, indicating that despite their major abundance on leptospiral membrane, their role in the binding of the host components assayed was minimal, either intrinsically or because of the upregulation of other receptors to “fill the niche” left by protein silencing.

### Interaction of the mutants with PLG and PLA formation

**Figure 3:**
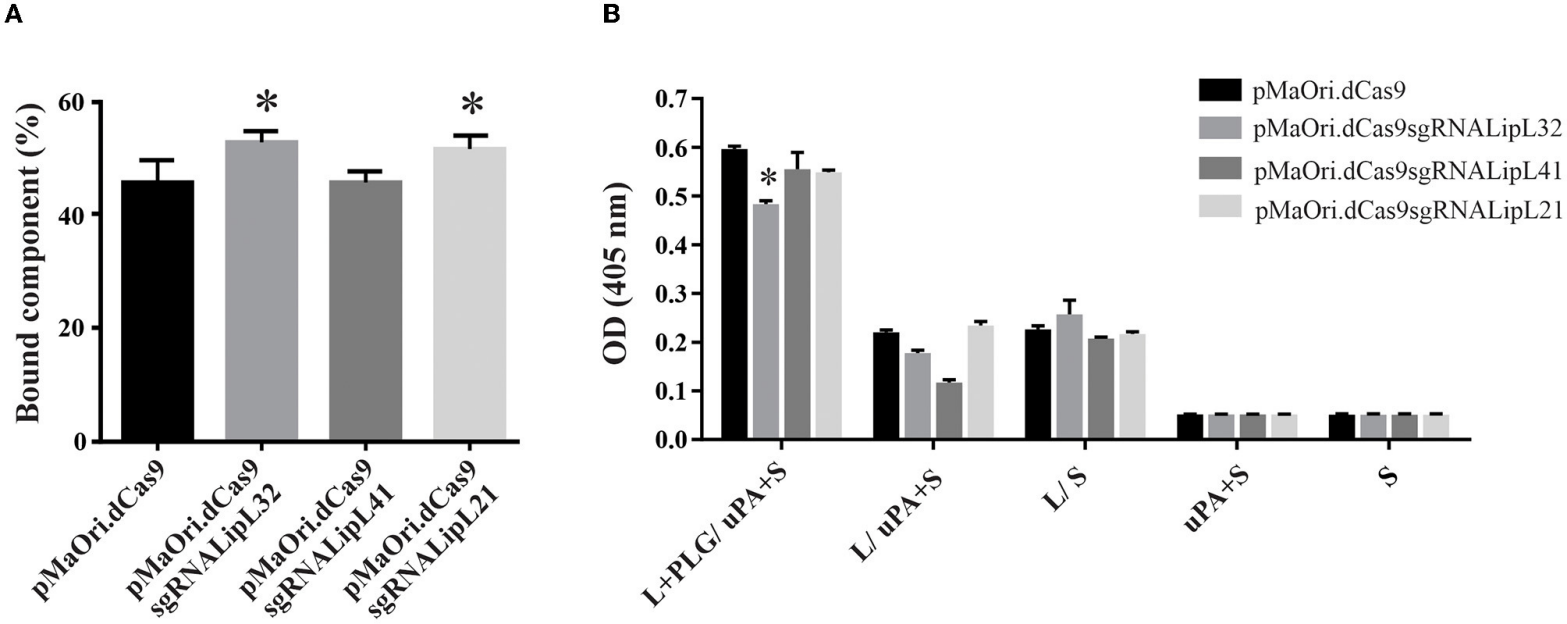
Interaction of leptospires with PLG and PLA formation. **(A)** Control or mutant *L. interrogans* cells were incubated with human plasminogen for binding evaluation by ELISA. The residual component mass in the supernatant was used to infer the mass bound to the leptospiral surface. **(B)** Leptospires (L) were incubated with human PLG, and cells were recovered by centrifugation and washed. Then, uPA along with the PLA substrate (S) was added. Controls lacking at least one component of the reaction or with no leptospires were included. Statistical analysis was performed using one-way ANOVA, followed by the Tukey post-test for pairwise comparisons of each group against control dCas9, and a *P*-value of < 0.05 (*) was considered to be statistically significant.

Control and knockdown leptospires were allowed to interact with human PLG (1 μg/mL), and the supernatant was immobilized in ELISA plates for component quantification, as described above. A significant increase in binding was observed for LipL32 and LipL21 mutants (*P* < 0.05), and the binding of LipL41 mutant to PLG was unaffected (Figure 3A). Possibly, the overall proteomic change as a direct result of gene silencing in the LipL32 and LipL21 mutants favors binding affinity for PLG. However, plasmin formation from the surface-bound PLG, after the addition of exogenous uPA and substrate, was mostly unaffected despite statistical significance (Figure 3B), strengthening again the notion that, despite their major abundance in the leptospiral membrane, their overall participation in PLG binding is minimal, which is consistent with the numerous leptospiral PLG-receptors described to date (Daroz et al., 2021).

### Evaluation of mutants' virulence in a hamster model

**Figure 4:**
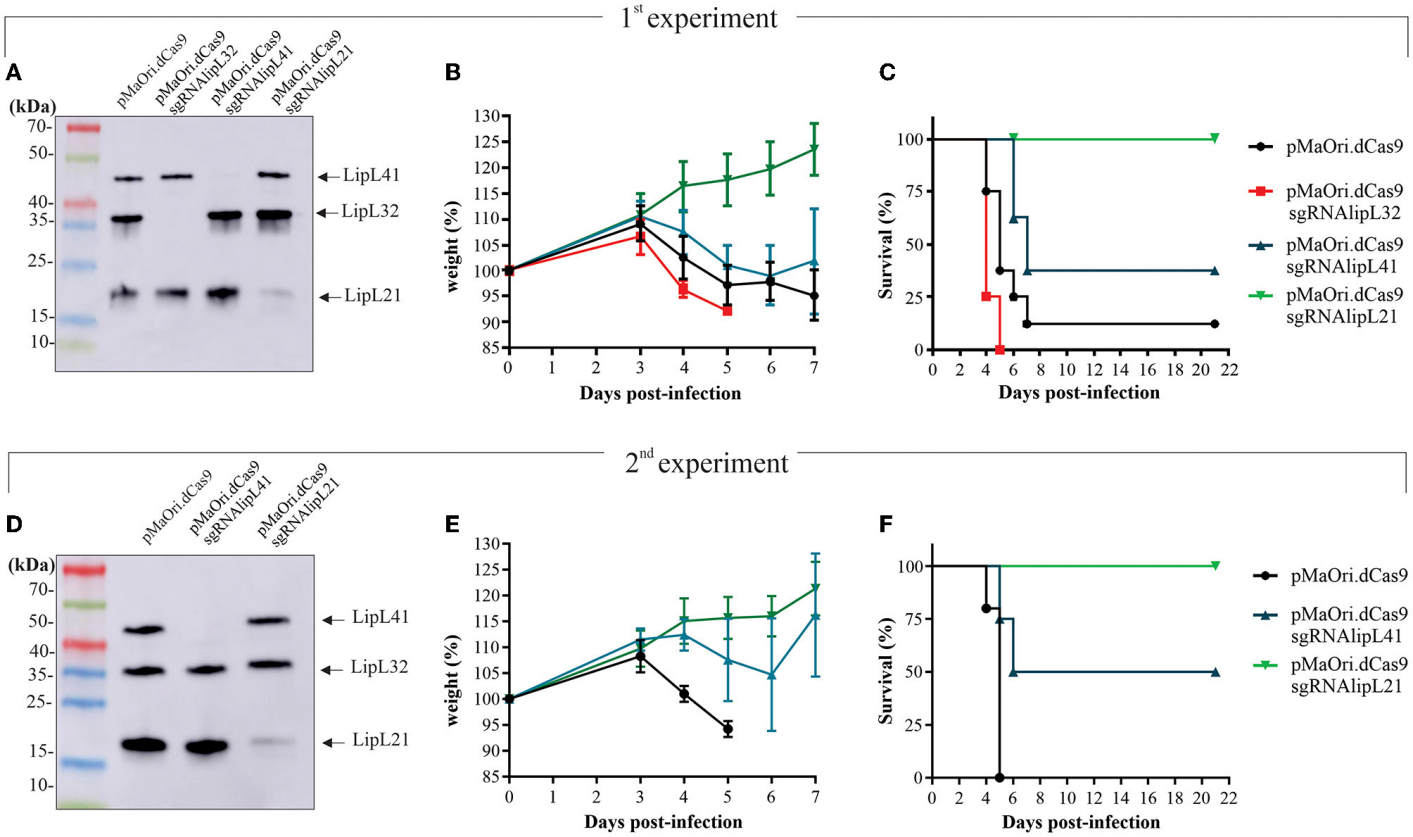
Virulence of leptospiral mutants in hamster model of infection. LipL32, LipL41, and LipL21 knockdown mutants and control cells containing pMaOri.dCas9 only were validated prior to animal infection **(A, D)**, and groups of recently weaned female Syrian gold hamsters (*n* = 8 in the first experiment and *n* = 4 in the second) were inoculated intraperitoneally with 5 × 10^7^ distinct and passage-synchronized leptospires. Each animal was monitored daily and weighed **(B, E)** for clinical signs of acute leptospirosis. Animals were humanely euthanized when weight loss (>10%) and/or additional clinical signs (blood on paws/nose/urogenital tract, lethargy) was observed **(C, F)**.

To assess mutants' phenotype in an animal model, each mutant or control pMaOri.dCas9-containing leptospires were re-validated by immunoblotting (Figure 4A) and then used to infect hamsters (*n* = 8) intraperitoneally. When endpoint criteria were met (Table 3), animals were humanely euthanized, and liver and kidney tissues were extracted. In this study, the acute disease is defined by the appearance of severe clinical symptoms in the first 7 days following experimental challenge. Tissue macerates were used to inoculate the medium without or with spectinomycin. All animals infected with the positive control, *L. interrogans* containing pMaOri.dCas9 only, exhibited clinical signs of acute leptospirosis, with prominent weight loss being observed first at day 4 (Figure 4B; Table 3). At day 7, seven out of eight animals (87.5%) had already met the endpoint criteria (Figure 4C), while for one animal, even though 10% weigh loss was observed at day 6, no evident clinical signs were observed, and this animal fully recovered.

**Table 3:** Animal experiment results.

	**Endpoints/total**	**Endpoint day**	**Average weight (%)** ^**a**^		
			**3**	**4**	**5**
**1st experiment**					
pMaOri.dCas9	7/8	4,4,5,5,5,6,7	109.1	102.6	97.1
pMaOri.dCas9sgRNAlipL32	8/8	4,4,4,4,4,4,5,5	106.5	96.4	92.52
pMaOri.dCas9sgRNAlipL41	5/8	6,6,6,7,7	110.4	107.4	101.1
pMaOri.dCas9sgRNAlipL21	0/8	6,6^*^	110.8	116.3	117.5
**2nd experiment**					
pMaOri.dCas9	5/5	4,5,5,5,5	108.3	101	94.2
pMaOri.dCas9sgRNAlipL41	2/4	5,6	111.5	112.4	107.6
pMaOri.dCas9sgRNAlipL21	0/4	–	109.7	115.0	115.7

^a^Compared to day 0 (100%).

^*^Animals taken for comparative purposes.

Consistent with earlier findings (Fernandes et al., 2021c), the virulence of LipL32 was found to be significantly higher than that of the control group (*P* < 0.05). By day 5, all animals in the LipL32 group had met the endpoint criteria (Table 3) and exhibited more severe clinical signs of leptospirosis, including a higher number of moribund animals compared to the pMaOri.dCas9 only-infected group. The LipL32 mutant-infected group displayed lower average weight at day 3 (*P* < 0.05) in comparison with the other groups. Contrarily, no apparent sign of disease or weight loss in the animals challenged with the LipL21 mutant strain was observed (*P* < 0.05) (Figures 4B), even though the protein was not completely silenced (>90% silencing), and two animals were euthanized at day 6 for comparing the bacterial burden in a “acute” time frame. For the LipL41 mutant-infected animals, no statistical difference was observed in comparison with the pMaOri.dCas9 only-infected group, and animals first met endpoint criteria at day 6 (contrasting with day 4 in control and LipL32 mutant groups). On day 7, five animals (62.5%) had already met endpoint criteria, and the remaining animals, which displayed slight weight loss, could fully recover. Individual weight measurements are presented in Supplementary Figure 4.

In a subsequent experiment, a new set of virulent leptospiral mutants was generated, validated (Figure 4D), and used to infect animals. Given that the increased virulence of the LipL32 mutant had already been established, only the LipL41 and LipL21 knockdown mutants were included in comparison with the control group infected with pMaOri.dCas9-containning leptospires. In this experiment, one set of animals from each group (*n* = 4) was euthanized on day 4 to compare the bacterial burden in target organs, while the other set (*n* = 5 or 4) was monitored until meeting the endpoint criteria.

The relative weight of dCas9 only-infected animals dropped at day 4 (Figure 4E; Table 3), consistent with all animals (*n* = 5) meeting endpoint criteria at day 5 (Figure 4F). In agreement with the first experiment, all LipL21 mutant-infected animals neither met endpoint criteria (*P* < 0.01) nor displayed weight loss, confirming the attenuation due to LipL21 silencing and the role of this protein in acute disease. The animals infected with the LipL41 mutant had delayed weight loss (Figure 4E; Table 3), and only 50% met endpoint criteria (*P* < 0.05).

### Mutant recovery from target organs and bacterial burden quantification

**Figure 5:**
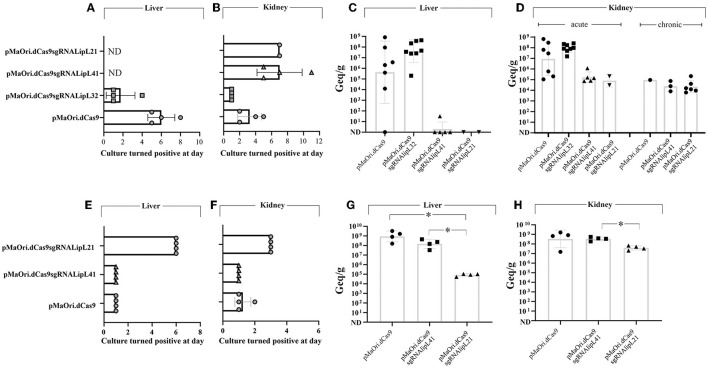
Leptospiral mutant isolation in a liquid medium and bacterial load quantification in target organs from infected animals. Kidney and liver from euthanized animals were macerated and homogenates used to inoculate liquid EMJH medium plus spectinomycin (recovery of only mutant cells), cultures were monitored daily regarding the presence of leptospires, and the day where they turned positive in the presence of antibiotics was recorded [**(A, B)**, first experiment, **(E, F)**, second one]. Organs were also used for total DNA extraction and bacterial burden quantification by quantitative PCR [**(C, D)**, first experiment, **(G, H)**, second one]. Bacterial loads are expressed in genome equivalents (Geq) per gram of tissue. Statistical analysis was performed using one-way ANOVA, followed by the Tukey post-test for pairwise comparisons and a *P*-value of < 0.05 (*) was considered to be statistically significant.

In the first experiment, kidneys and livers from four animals that were euthanized in the acute time frame were used to prepare homogenates followed by inoculation in a liquid medium with or without spectinomycin. Cultures were monitored daily for the presence of motile leptospires, and a positivity score was given depending on their presence and abundance in the medium with antibiotics (mutant cells only). All kidney and liver cultures from the pMaOri.dCas9 only and LipL32 mutant were positive for the presence of leptospires; interestingly and consistent with the increased virulence of LipL32 mutant, positive cultures were recorded as soon as day 1 for all cultures from kidney and three out of four liver homogenates of this group (Figures 5A, B; Supplementary Figure 5). LipL32 mutant-derived cultures also reached higher cell densities earlier than those from the control group. In all cases, similar growth profile was observed in cultures with or without spectinomycin, indicating that most of the cells were still harboring the plasmids for gene silencing.

Liver macerate cultures from LipL41 and LipL21 mutants were negative for leptospires (Figure 5A), most probably because of the later time point when these animals met endpoint criteria (for LipL41 mutant-infected animals) or when animals were taken for “acute time point” comparative purposes (LipL21 mutant). Although kidney cultures were positive, they first turned positive at days 5 and 7 for LipL41 and LipL21 mutant groups (Figure 5B; Supplementary Figure 5), respectively, indicating lower bacterial burden and/or cell viability of these mutants in regard to pMaOri.dCas9 only control group.

The results of bacterial quantification in the liver (Figure 5C) and kidney (Figure 5D) confirmed the higher bacterial load in animals infected with LipL32 mutant, with a geometric mean of 4 × 10^7^ in the liver, compared to 4.3 × 10^5^ in the control animals, and 9.1 × 10^7^ in the kidneys, compared to 8.9 × 10^6^ in control animals. Because of animal variability in the pMaOri.dCas9 group, statistical significance was not achieved between these groups. Liver loads were reduced or not found in LipL41 mutant- and LipL21 mutant-infected animals. Kidney loads in the “acute time frame” were reduced in these groups, with an average of 1.7 × 10^5^ and 7.9 × 10^4^ for LipL41 and LipL21 mutant-infected animals, contrasting with 8.9 × 10^6^ in pMaOri.dCas9 only-infected group. Kidney loads in the surviving animals were similar between groups (Figure 5D).

When acute time frame-synchronized animals were euthanized (day 4) in the second challenge experiment, liver macerates were positive for LipL41 mutant and pMaOri.dCas9 group at day 1 of growth, the latter reaching higher cell densities earlier, and in contrast, growth was observed at day 6 for the LipL21 mutant group (Figure 5E). Cultures from kidney macerates turned positive at day 1 for all LipL41 mutant group and for three out of four in pMaOri.dCas9 control since the presence of leptospires was only observable at day 2 in one of these cultures (Figure 5F). Compatible with previous results, LipL21 mutant cultures from kidney homogenates were positive at day 3 of incubation (Figure 5F). Bacterial quantification results agreed with macerates' cultures, whereas liver loads were drastically reduced in the LipL21 mutant-infected animals, which displayed an average of 8.4 × 10^4^ Geq/g of tissue, contrasting with 9 × 10^8^ (*P* < 0.05) and 1.5 × 10^8^ (*P* < 0.05) for dCas9 only and LipL41 mutant-infected animals, respectively (Figure 5G). Lesser extent contrasts were found in the kidneys, with the similar number of leptospires found in the dCas9 only and LipL41 mutant groups (3.4 × 10^8^) and 3.9 × 10^7^ in the LipL21 mutant-infected animals (Figure 5H).

### Demonstration of phenotype stability *in vivo* and *in vitro*

**Figure 6:**
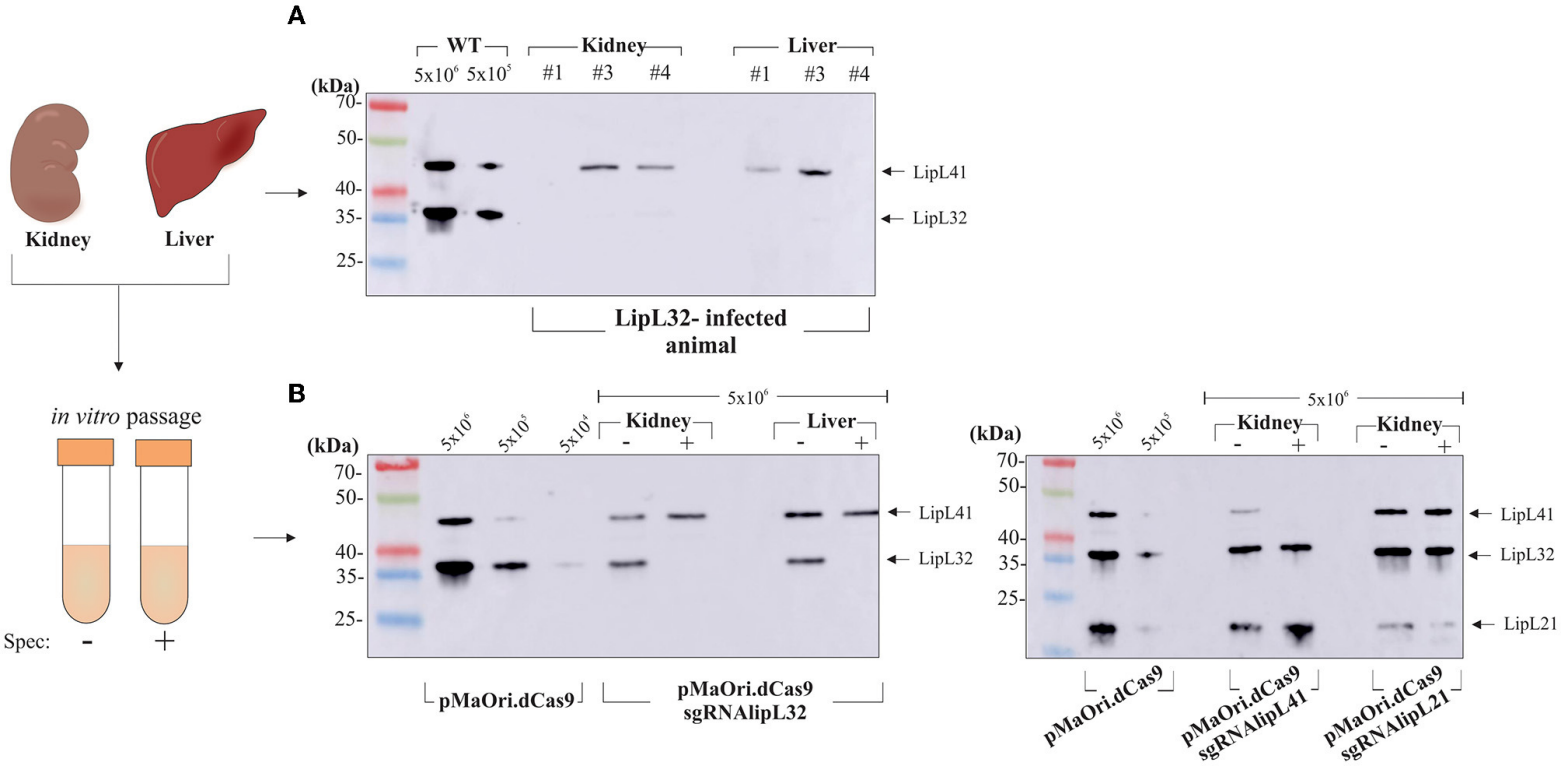
*In vivo* and *in vitro* silencing phenotype stability. **(A)** Protein silencing in leptospires sheltered at the target organs. Tissue macerates from kidney and liver sections were centrifuged at low speed, and the supernatant was carefully recovered. After centrifugation, pellets were washed and resuspended for preparation of cell lysates, which were evaluated by immunoblotting. Different numbers of control leptospires were also used for comparison purposes. **(B)** Target protein expression in leptospires cultured out of EMJH medium after inoculation of organ macerate, with (+) or without (–) spectinomycin (40 μg/mL), was also assessed. Bacteria were recovered from the medium and washed, and suspensions were then counted. Whole-cell lysates corresponding to 5 × 10^6^ leptospiral cells were evaluated by immunoblotting. For comparative purposes, various amounts of wild-type (WT) leptospires or leptospires containing the control pMaOri.dCas9 were included in the experiment.

Exploiting the higher bacterial burden in target organs of LipL32-infected animals, silencing profiles were assessed directly from the tissue macerates from three animals, normalized by volume. Lysates were separated by SDS-PAGE and proteins were evaluated by immunoblotting. As depicted in Figure 6A, when leptospires were detected in the macerates by co-incubation with anti-LipL41 and anti-LipL32, only the LipL41 protein could be detected, contrasting with intense LipL32 reactivity in wild-type control at a different number of leptospires per well, directly demonstrating the plasmid upkeep *in vivo*, and by consequence, gene silencing. It is worth mentioning that leptospires from the pMaOri.dCas9 only-infected organs could not be detected in the lysates (data not shown), most probably due to lower bacterial loads.

Tissue macerates were also used to inoculate EMJH medium (50 μL in 5 mL) with (+) or without (–) spectinomycin, the latter to recover only mutant cells. For the acute time frame, cell lysates from knockdown or control *Leptospira* after one *in vitro* passage were compared. Regarding the mutants isolated from the kidney and liver (in the case of LipL32 mutant), only a minor target protein expression band was observed when leptospires grew in medium without antibiotic (Figure 6B), whereas the presence of the antibiotic resulted in the retrieval of only mutant cells. Kidney macerate cultures from the LipL41 and LipL21 mutant-infected animals confirmed the silencing in the presence of spectinomycin, with only minor target protein reappearance when no antibiotic was added to the medium (Figure 5B), confirming phenotype stability.

## Discussion

Extensive literature exists that focuses on understanding how pathogenic *Leptospira* interact with their hosts (reviewed by Daroz et al., 2021). This was primarily accomplished through analyzing the entire genome sequences, mainly of *L. interrogans*, resulting in the creation of recombinant proteins that have been extensively studied to determine their interaction with host components. These studies have shed light on leptospiral surface-exposed proteins that may be involved in colonization (Barbosa et al., 2006; Pinne et al., 2010; Vieira et al., 2010b; Fernandes et al., 2012; Siqueira et al., 2013; Teixeira et al., 2015; Silva et al., 2016), invasion (Vieira et al., 2010a; Oliveira et al., 2013; Pereira et al., 2017; Santos et al., 2018; Takahashi et al., 2021), and immune evasion processes (Verma et al., 2006; Stevenson et al., 2007; Barbosa et al., 2010; Castiblanco-Valencia et al., 2012, 2016; Siqueira et al., 2016; Cavenague et al., 2019). Although *in vitro* experiments have yielded promising results, researchers often encounter frustration when the antigens fail to be protective in vaccine tests or when mutants still prove to be virulent in animal infection models.

The development and application of CRISPRi allowed easy, specific, and one-step generation of knockdown mutants in several pathogenic *Leptospira*, including the newly described *L. sanjuanensis* (Fernandes et al., 2022) and *L. borgpetersenii* (Fernandes et al., unpublished data), making it feasible to evaluate virulence factors in an animal model (Fernandes et al., 2021c). This scenario has brought the spotlight back to the evaluation of the role of major OMPs in leptospiral pathogenicity, which could ultimately guide the rationale for the generation of subunits and/or chimeric vaccines.

LipL32 protein was first characterized by Haake et al. (2000), when the authors concluded that this protein could be relevant to *Leptospira* pathogenesis. However, a transposon mutant was still capable of causing both acute and chronic disease as effectively as the wild-type strain (Murray et al., 2009). Recently, a knockdown LipL32 mutant obtained by CRISPRi was re-evaluated in a hamster model, and the results consistently suggested that the silencing of this protein leads to a more perceptible virulence phenotype, with the presentation of early (and more severe) symptoms and higher liver bacterial burden (Fernandes et al., 2021c).

To further confirm this interesting finding, a new batch of LipL32 knockdown mutant was obtained and used to infect hamsters. Accordingly, we demonstrated that LipL32 silencing results in more virulent *Leptospira* cells, culminating in early and more severe symptom presentation in hamsters, accompanied by higher liver and kidney loads. Remarkably, taking advantage of this higher organ load, we could demonstrate LipL32 silencing directly from organ macerates, proving that silencing is maintained *in vivo*, at least in the “acute” time frame.

The interaction of LipL32 mutant with host components was unaffected, except for a slight increase in PLG binding, which could be explained by minor participation of this protein in *Leptospira–*host interactions or by a compensation mechanism due to the upregulation of proteins with more affinity for host ligands to fill the niche left by LipL32 silencing. Our results agree with the ones from Murray et al. (2009) that showed that the LipL32 mutant binding to laminin was unaffected. Previous whole-cell proteome analysis performed on the LipL32 mutant cells showed an overall change in protein abundance profile, with 84 proteins being upregulated, including the now well-established cooperative virulence factors LigA and LigB, which could help explain the increased virulence of this mutant. In addition, LipL41 and LipL21 proteins were significantly up- and downregulated in the LipL32 mutant, respectively (Fernandes et al., 2021c). However, these differential expressions were not perceivable in our immunoblots.

LipL41 protein has been widely studied since it was first described, including its interaction with host molecules (Takahashi et al., 2021). Recombinant LipL41 protein could efficiently bind to elastin, collagen IV, laminin, E-cadherin, cellular fibronectin, PLG, and fibrinogen. In our results, the LipL41 knockdown mutant displayed slight and significant decreased binding to E-cadherin and fibrinogen (but fibrin clot formation was unaffected), whereas an increase was observed for collagen IV binding.

When a transposon mutant for LipL41 (*L. interrogans* serovar Pomona) was assessed in a hamster model of infection, it was still virulent as no animals survived the infection (King et al., 2013). Our animal infection results indicated that, even though the LipL41 knockdown mutant was still virulent, some animals could still survive the lethal challenge, with less severity observed in those that succumbed to infection and later endpoint dates, suggesting that a slight attenuation in virulence could occur by LipL41 silencing in *L. interrogans* strain Fiocruz L1-130. It is possible that attenuation could be more distinguishable by different infection routes or inoculum sizes. By comparing liver and kidney loads in time-synchronized animals, only a minor reduction in liver burden was observed in comparison with control leptospires containing only the pMaOri.dCas9 plasmid.

Supported by the data that LipL41 elicited a synergistic protection profile along with the porin OmpL1 against lethal challenge in hamsters (Haake et al., 1999), a double knockdown mutant was pursued but no colonies could be recovered; silencing of OmpL1 alone, in both pathogenic *L. interrogans* and saprophytic *L. biflexa*, was also not possible, suggesting the essentiality of OmpL1 protein.

OmpL1 protein is a unique porin encoded by *Leptospira* spp., and its topological model contains ten amphipathic transmembrane β-strands (Haake et al., 1993; Shang et al., 1995). The outer face of the β-strand is characterized by its hydrophobic nature, facilitating interactions with the lipid bilayer. On the contrary, the inner face exhibits hydrophilic properties, allowing it to interact with the aqueous pore within the protein (Haake et al., 1993). The porin superfamily is composed of proteins that form membrane-spanning, water-filled channels permitting the diffusion of hydrophilic molecules into the periplasmic space (Jeanteur et al., 1991). Some exception can occur since it was previously described that outer membrane protein OmpW from *E. coli* (Hong et al., 2006) and OprG from *Pseudomonas aeruginosa* (Touw et al., 2010) form an eight-stranded beta-barrel with the hydrophobic channel. Major outer membrane porin (PorB), which mediates ion exchange between *Neisseria gonorrhoeae* and the environment (Chen and Seifert, 2014), was shown to be essential to these bacteria, likely due to its ability to allow nutrients access to the periplasm (Haines et al., 1988). In this sense, OmpL1 protein could be mechanistically involved in nutrient uptake or membrane integrity in *Leptospira* spp.

LipL21 has been shown to be a surface-exposed protein in *L. interrogans* by previous studies (Cullen et al., 2003; Techawiwattanaboon et al., 2021). Seeking to explore the potential role of native LipL21 in virulence and host–pathogen interactions, a knockdown LipL21 mutant was obtained. LipL21 mutant *Leptospira* binding to host components was mostly unaltered, with only a modest increase in laminin and PLG binding; however, when used to infect hamster, the LipL21 mutant was consistently unable to cause acute disease.

Evaluation of target organ loads in time-synchronized “acute phase” animals revealed remarkably decreased liver loads (~4 logs) in LipL21 mutant-infected animal in comparison with the control, whereas kidney loads were only slightly reduced. These results agree with the animal phenotype displayed by the LigA/B knockdown mutant (Fernandes et al., 2021c), which also causes asymptomatic infections in hamster with renal colonization and with drastically reduced liver loads, hinting that bacterial hepatic concentration in the early time point of infection could be a feature of acute leptospirosis, which is also in agreement with the higher liver loads of the more severe LipL32 mutant.

There are several mechanisms that could explain this virulence attenuation. It has been previously shown that LipL21 protein is tightly bound to the leptospiral peptidoglycan, protecting it from degradation into muropeptides, thereby blocking signaling through nucleotide oligomerization domain (NOD)-like receptors (NOD1 and NOD2) proteins, potentially helping *L. interrogans* to circumventing innate host responses (Ratet et al., 2017). Even though both interactions with periplasmic peptidoglycan and surface-exposure could be mutually exclusive features due to LipL21 size, it has been proposed that this protein could coexist in two forms, one residing in the periplasm and another free in the outer membrane (Ratet et al., 2017), similar to the Lpp protein from *E. coli* (Cowles et al., 2011).

In addition, Kumar et al. (2022) demonstrated that recombinant LipL21 interacts with complement negative regulator C4bp and FH, favoring serum resistance, and displays *in vitro* nuclease activity toward neutrophil extracellular trap (NET); LipL21 protein can modulate neutrophil function by inhibiting myeloperoxidase (MPO) activity (Vieira et al., 2018).

The virulence phenotype of double or triple mutants remains to be evaluated since the multiple mutants obtained in this study displayed genetic instability during *in vitro* cultivation. The usage of different promoters for different sgRNA cassettes for avoiding recombination or pursuing gene silencing in the genetic background of a recently obtained *L. interrogans* knockout for LipL32 (Fernandes and Nascimento, 2022) are interesting strategies and are being evaluated by our group.

In conclusion, we demonstrated augmented bacterial virulence as a result of LipL32 silencing, and a subtle attenuation of acute disease presentation in the LipL41 mutant. LipL21 silencing resulted in the inability of leptospires to cause acute disease, even though they could still colonize the kidney. The participation of OmpL1 porin in virulence and host–pathogen interaction could not be determined, since its silencing was lethal to both saprophyte and pathogenic *Leptospira*, indicating its essentiality to leptospiral basic biology. CRISPRi is now a well-established genetic tool for exploring leptospiral pathogenic mechanisms that will increase our knowledge of this fascinating microorganism and add new pieces to the complex puzzle of leptospirosis.

## Data availability statement

The original contributions presented in the study are included in the article/Supplementary material, further inquiries can be directed to the corresponding authors.

## Ethics statement

The animal study was reviewed and approved by Animal Care and Use Committee at Instituto Butantan.

## Author contributions

LGVF, AFT, and ALTON: literature revision, manuscript preparation, and experimental design. LGVF and AFT: experimental manipulation. LGVF: figures. All authors reviewed and approved the manuscript, participated in the literature revision, discussion, and preparation of manuscript.

## Supplementary material

Supplementary Figure 1 Phenotype instability in the double LipL32/LipL41 mutants. After colony formation, distinct colonies (a to d) were selected and grown in a liquid medium plus spectinomycin (P1) and then re-inoculated in the same medium and monitored until mid-log phase (P2). Cell lysates from both P1 and P2 cultures were evaluated by immunoblotting with anti-LipL32, anti-LipL41, and anti-LipL21 antisera.

Supplementary Figure 2 OmpL1 silencing is lethal to *L. biflexa* cells. Plasmids pMaOri.dCas9 alone or containing a sgRNA cassette targeting the *ompL1* gene were delivered by conjugation to *L. biflexa* cells. Colonies recovered in EMJH plates plus spectinomycin were counted in two independent experiments **(A)**, and representative images of the plates were taken **(B)**.

Supplementary Figure 3 Fibrinogen binding and fibrin clot inhibition by leptospires**. (A)** *L. interrogans* containing empty pMaOri.dCas9 (control) or knockdown mutants to LipL32 (pMaOri.dCas9sgRNAlipL32), LipL41 (sgRNAlipL41), and LipL21 (sgRNAlipL21) were incubated with human fibrinogen for binding evaluation by ELISA. Reactivity was revealed with o-phenylenediamine substrate, and the residual component mass in the supernatant was used to infer the mass bound to the leptospiral surface. Statistical analysis was performed using one-way ANOVA, followed by the Tukey post-test for pairwise comparisons of each group against control dCas9, and a *P*-value of < 0.05 (^*^) was considered to be statistically significant. **(B)** Leptospires were resuspended in a human fibrinogen solution (1 mg/mL) to a final OD_420nm_ of 0.5. After 2 h incubation at 37°C, 90 μL of the incubation reactions were added to each well of a microdilution plate and mixed with 10 μL of 0.5 U/mL human thrombin. Experiments were performed with six replicates, and controls with no leptospires (maximal fibrin clot formation) or no thrombin (no fibrin clot formation) were included. Plates were read at 600 nm at 2-min intervals. The results are expressed in percentage of clot formation, where clot turbidity in the control with no leptospires was considered 100%.

Supplementary Figure 4 Daily measurements of hamsters' weight. Animals' weight from the first and second experiments were recorded daily. For each animal, individual weight displayed at day 0 was considered 100%. LipL32 mutant was not included in the second experiment.

Supplementary Figure 5 Daily observation and positivity scores from medium inoculated with tissue macerates. Cultures containing spectinomycin (retrieval of only mutant leptospires) were monitored daily for the presence and abundance of leptospires, and positivity scores were recorded. 1: 1 to 10 leptospires per field; 2: 10 to 100; 3: >100 leptospires per field. LipL32 mutant was not included in the second experiment.
