# Supplementary figures and images for: Evaluation of Leptospira interrogans knockdown mutants for LipL32, LipL41, LipL21, and OmpL1 proteins
Source: Front Microbiol. 2023 Jun 23;14:1199660. doi: 10.3389/fmicb.2023.1199660 (PMC10326724; doi:10.3389/fmicb.2023.1199660)

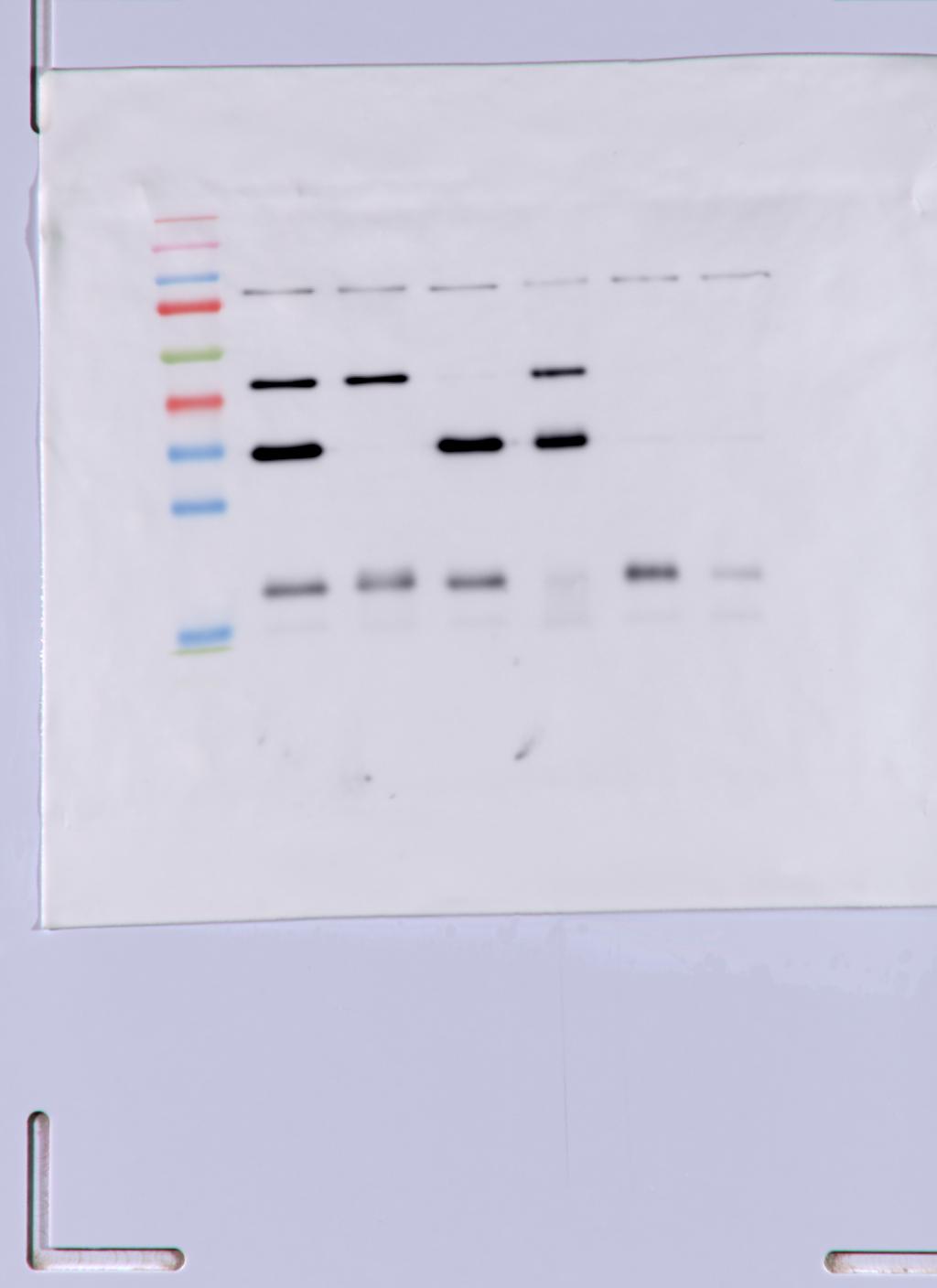

Supplement: Supplementary file 1 [file Data_Sheet_1.ZIP › Figure 1 Full Western.jpg]

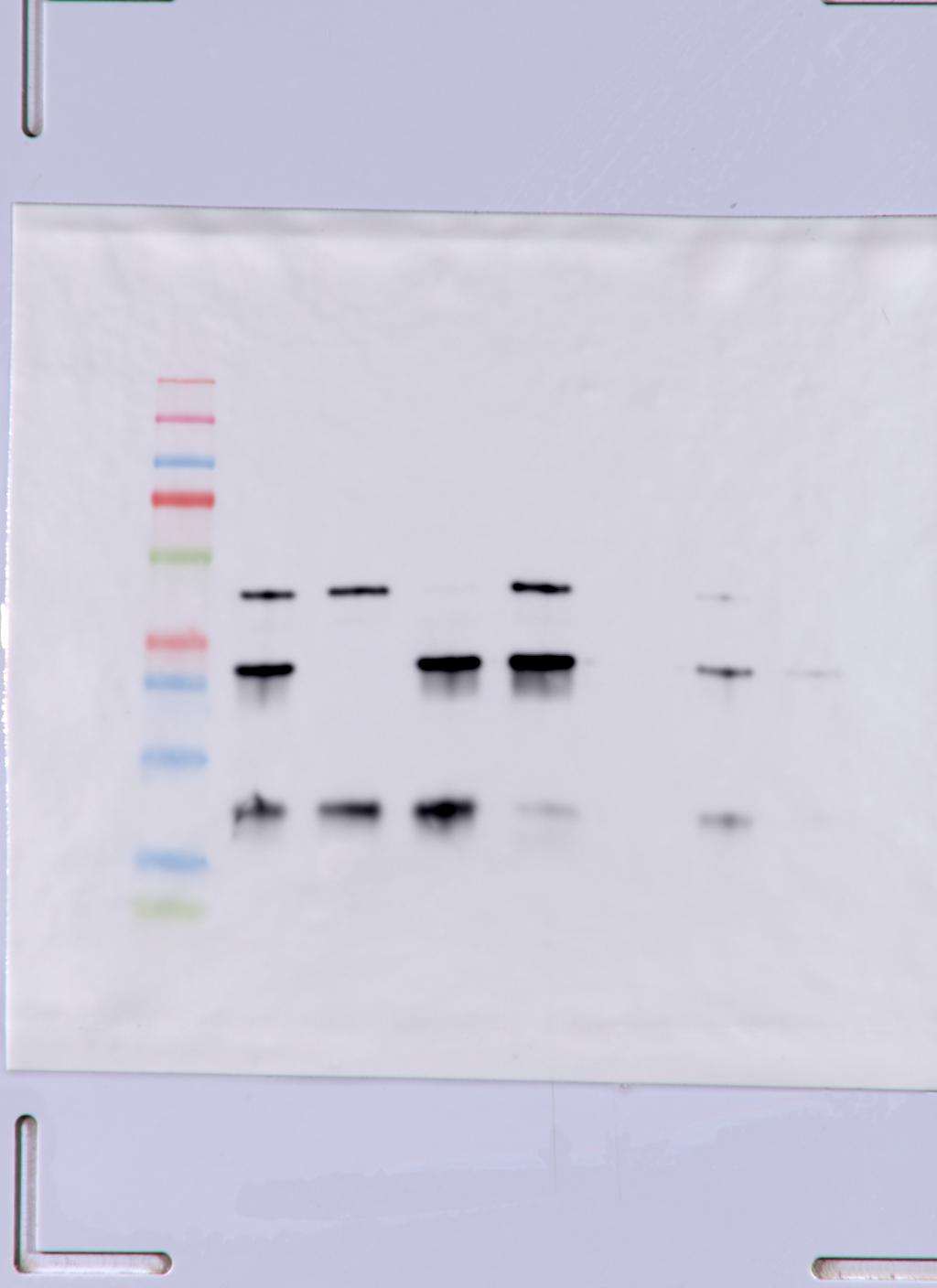

Supplement: Supplementary file 1 [file Data_Sheet_1.ZIP › Figure 4 Panel A Western.jpg]

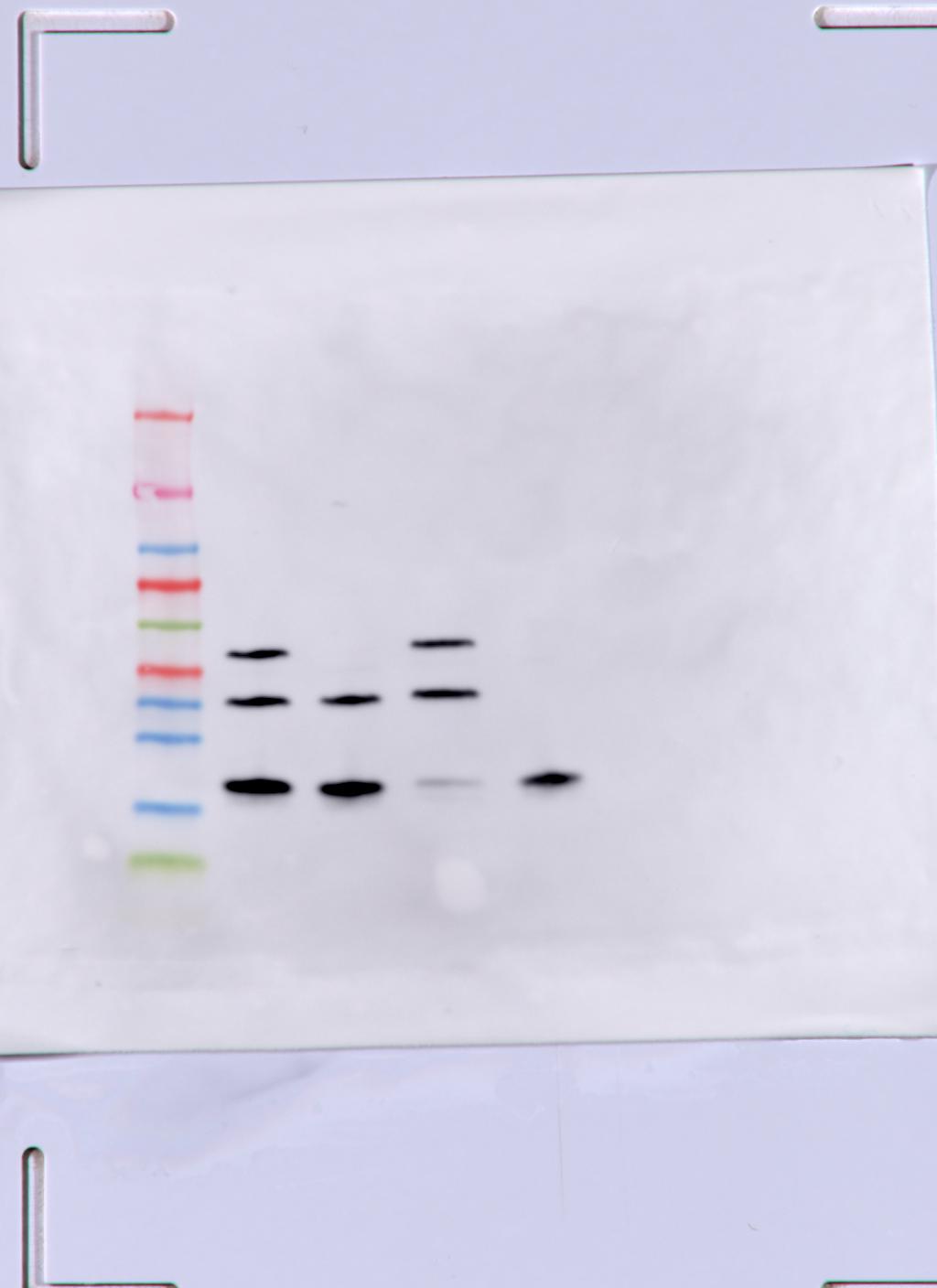

Supplement: Supplementary file 1 [file Data_Sheet_1.ZIP › Figure 4 Panel D Western.jpg]

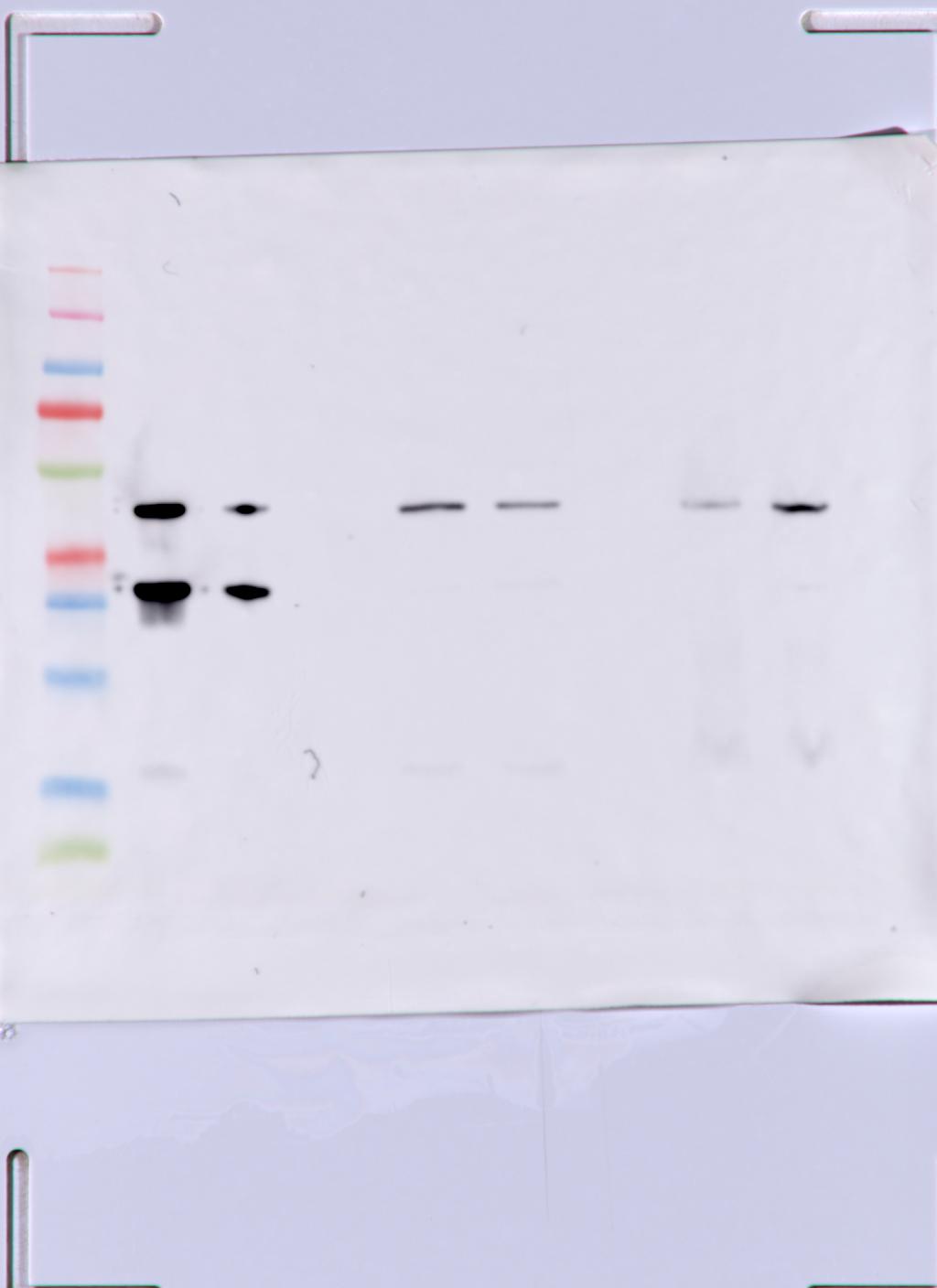

Supplement: Supplementary file 1 [file Data_Sheet_1.ZIP › Figure 6 Panel A Western.jpg]

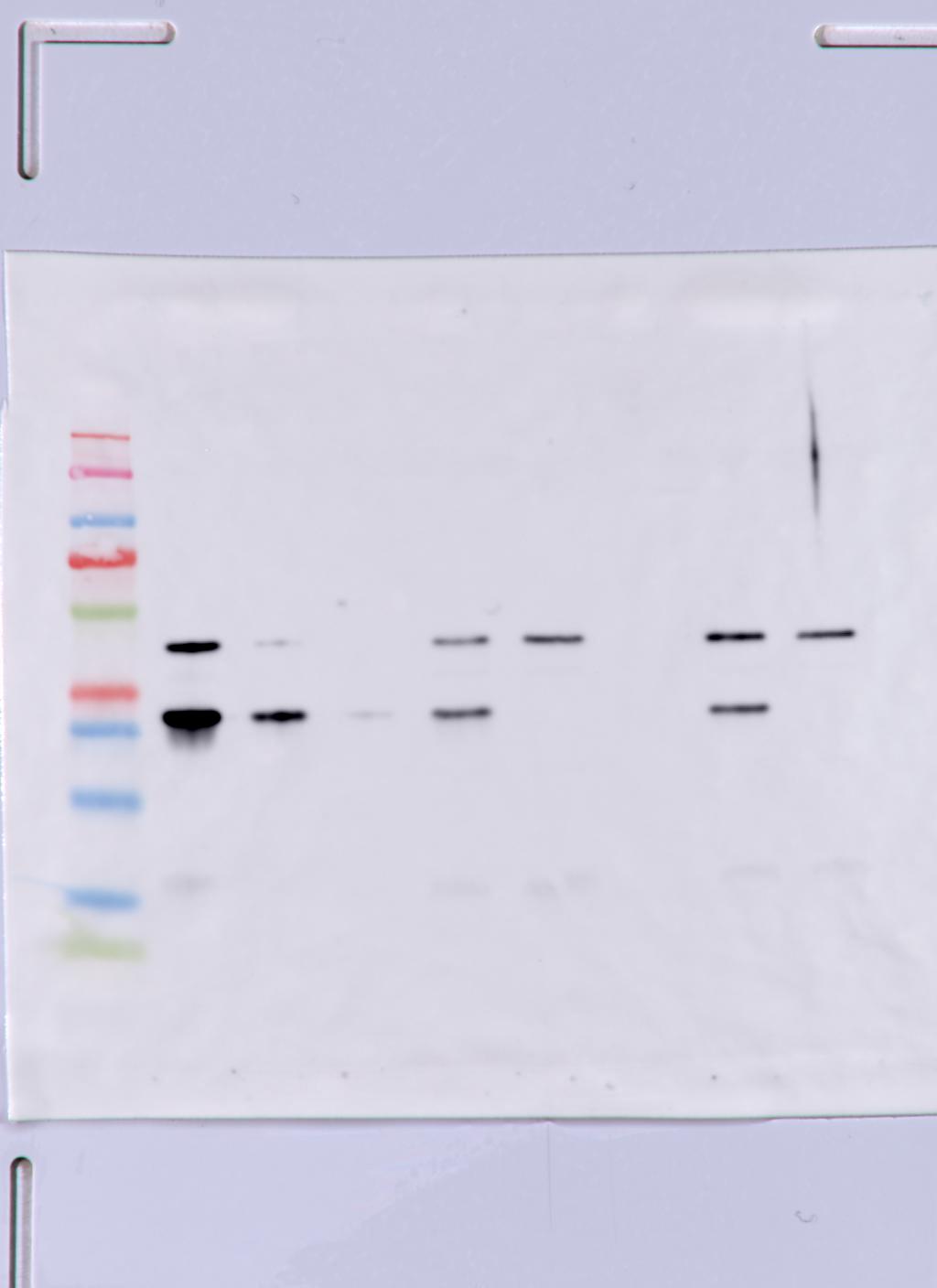

Supplement: Supplementary file 1 [file Data_Sheet_1.ZIP › Figure 6 Panel B Western.jpg]

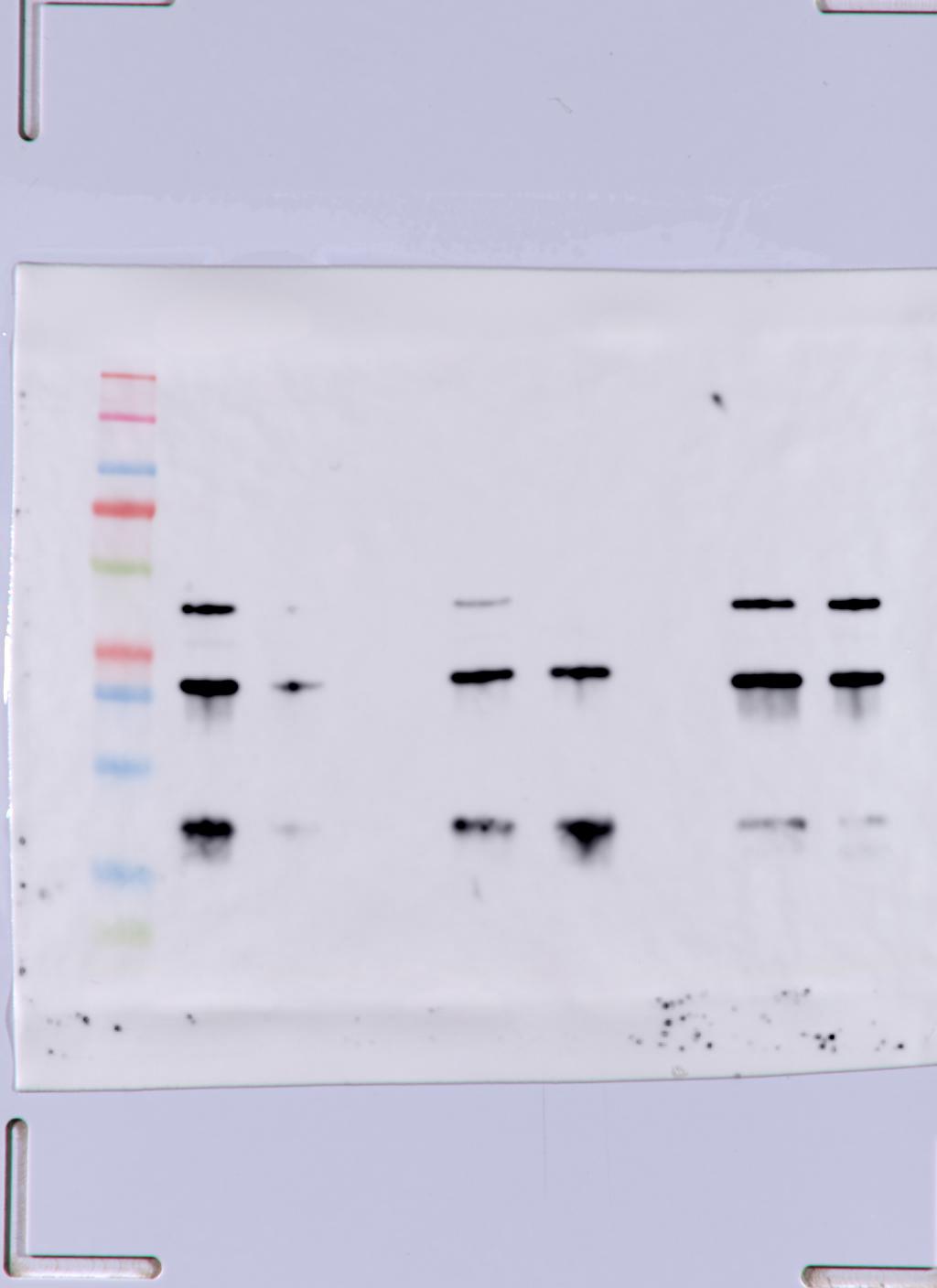

Supplement: Supplementary file 1 [file Data_Sheet_1.ZIP › Figure 6 Panel C Western.jpg]

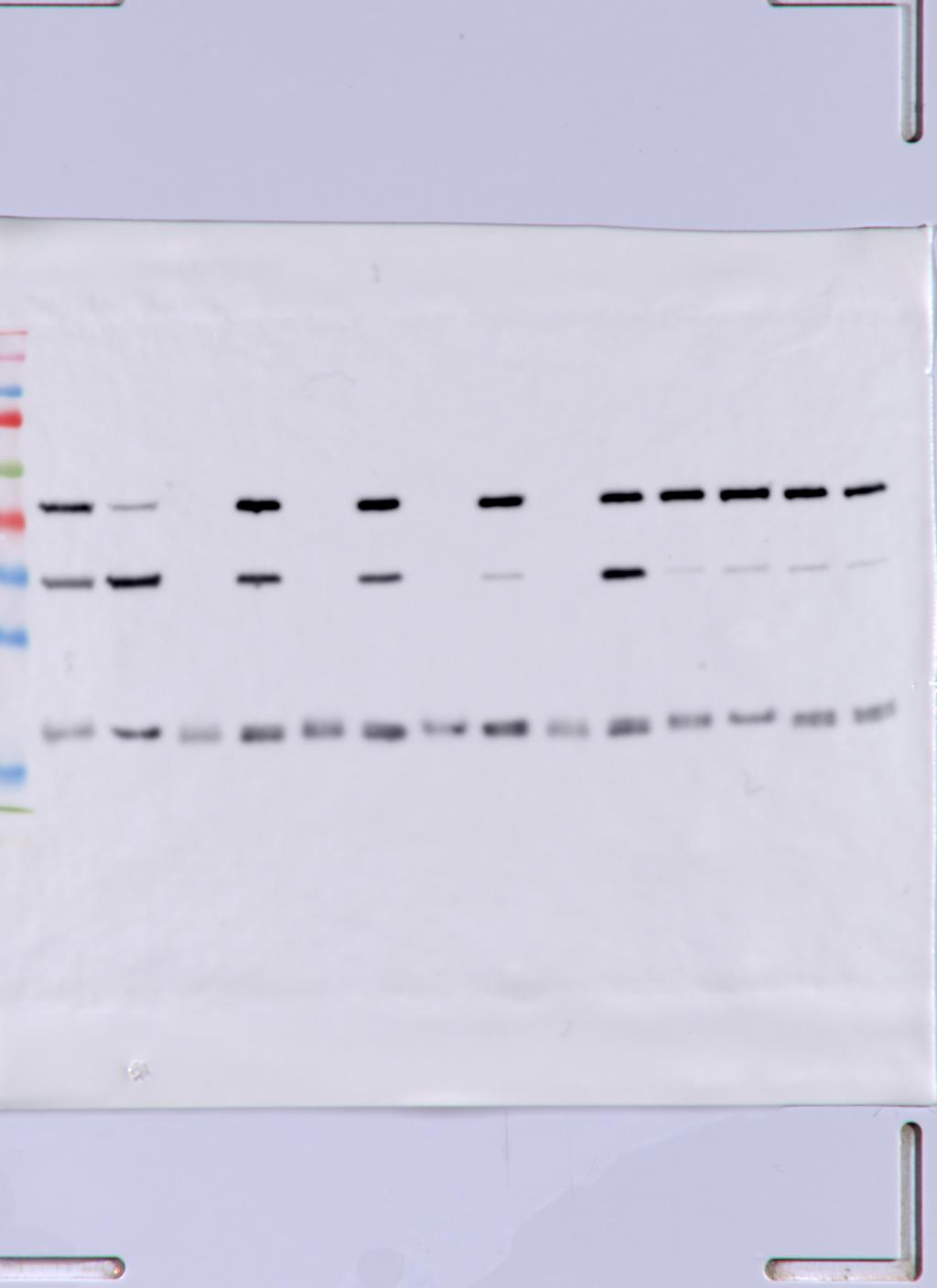

Supplement: Supplementary file 1 [file Data_Sheet_1.ZIP › Supplementary 2 Western.jpg]

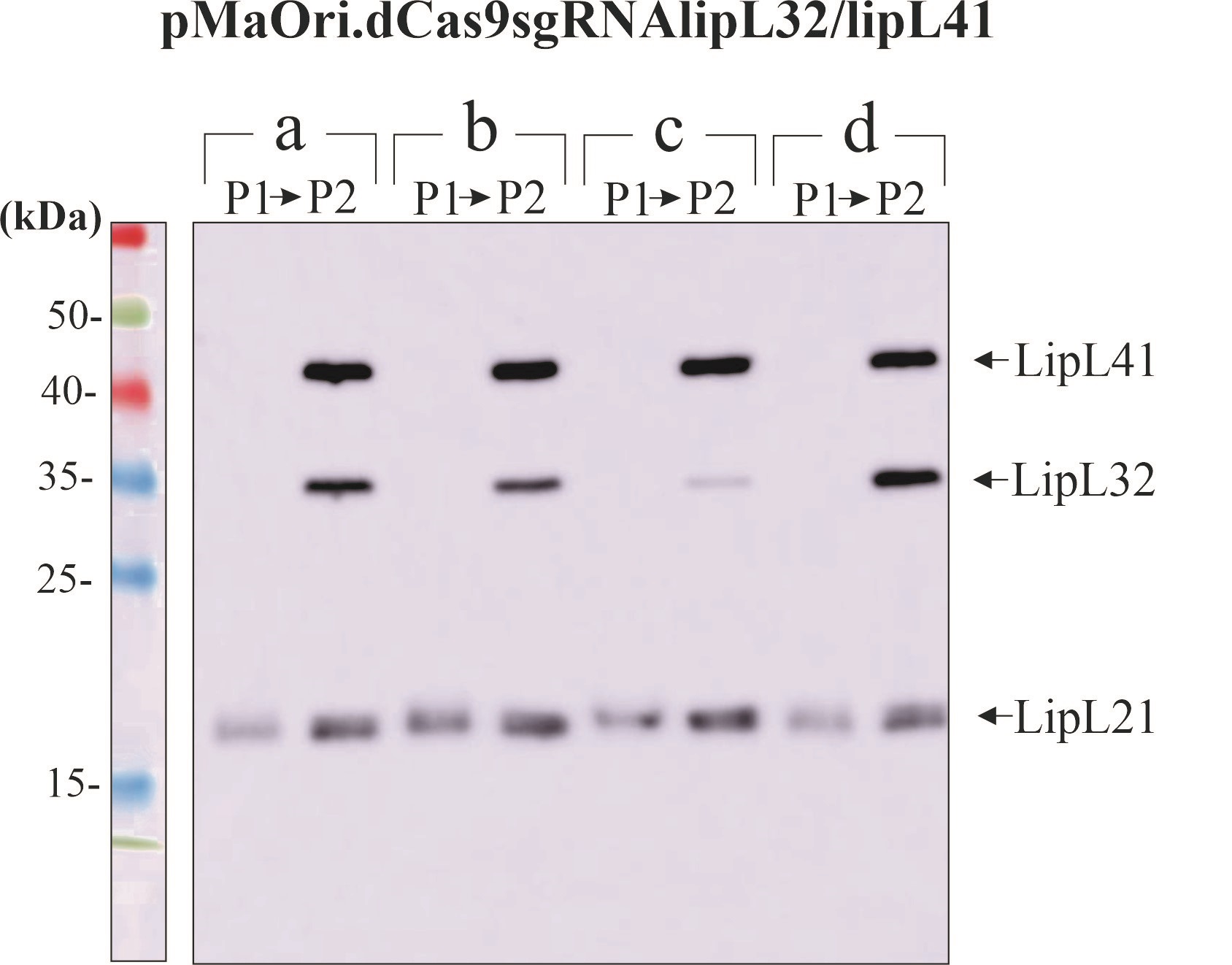

Supplement: Supplementary Figure 1 — Phenotype instability in the double LipL32/LipL41 mutants. After colony formation, distinct colonies (a to d) were selected and grown in a liquid medium plus spectinomycin (P1) and then re-inoculated in the same medium and monitored until mid-log phase (P2). Cell lysates from both P1 and P2 cultures were evaluated by immunoblotting with anti-LipL32, anti-LipL41, and anti-LipL21 antisera. [file Image_1.jpg]

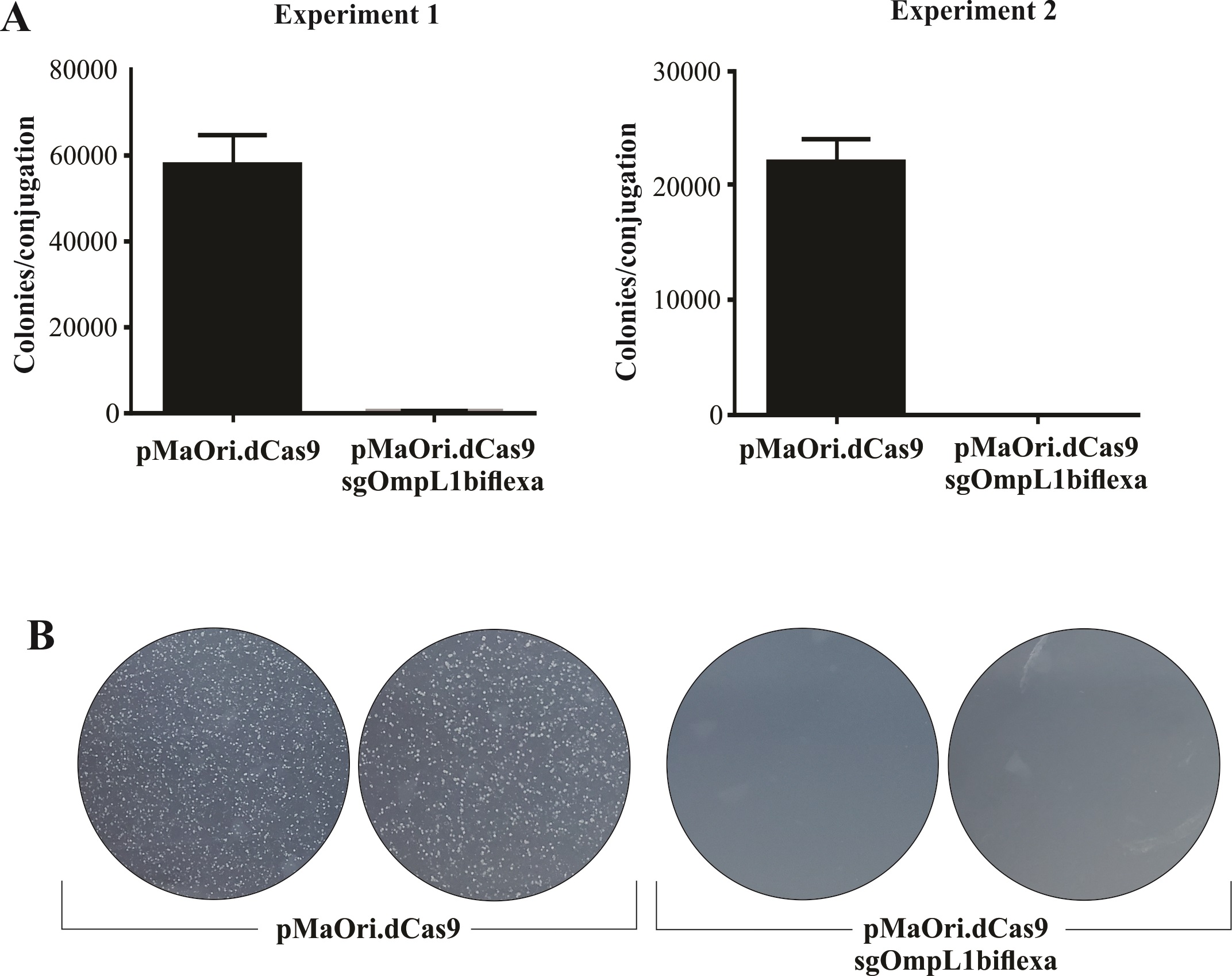

Supplement: Supplementary Figure 2 — OmpL1 silencing is lethal to L. biflexa cells. Plasmids pMaOri.dCas9 alone or containing a sgRNA cassette targeting the ompL1 gene were delivered by conjugation to L. biflexa cells. Colonies recovered in EMJH plates plus spectinomycin were counted in two independent experiments (A), and representative images of the plates were taken (B). [file Image_2.jpg]

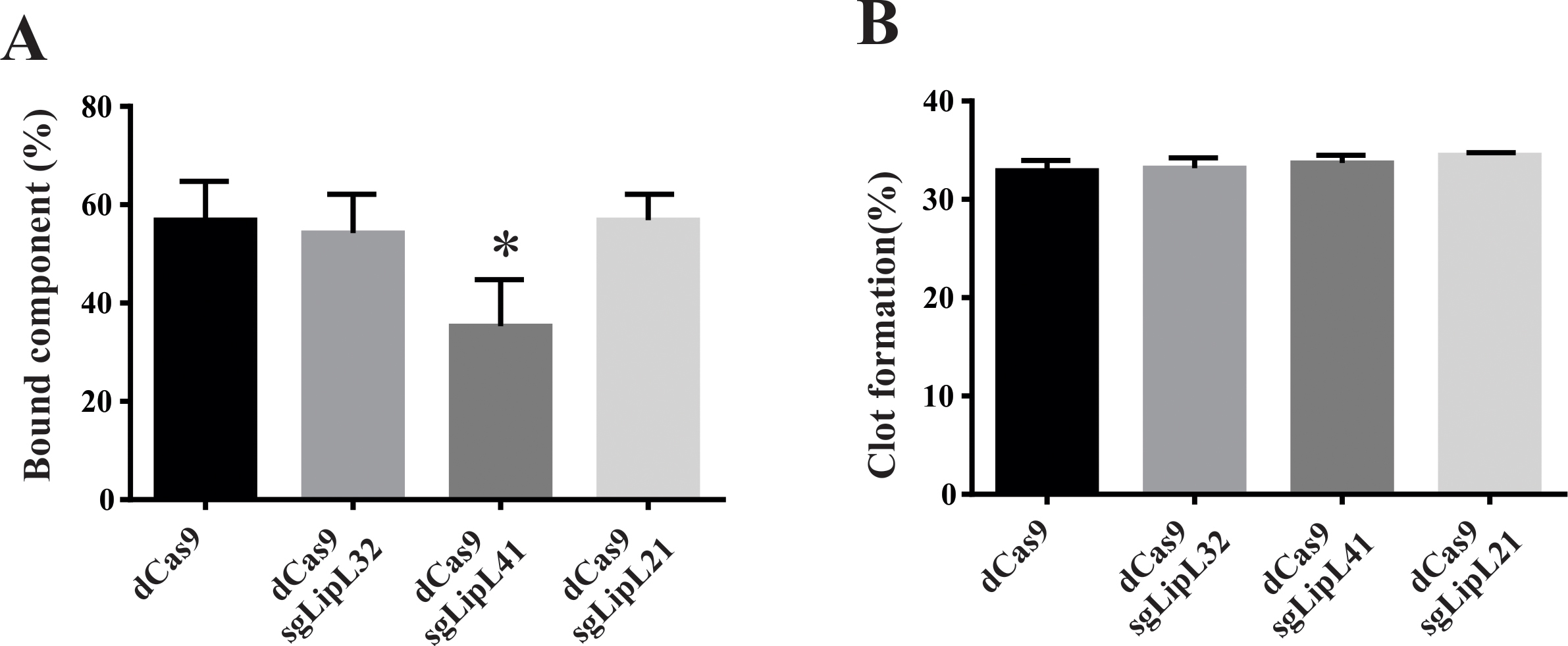

Supplement: Supplementary Figure 3 — Fibrinogen binding and fibrin clot inhibition by leptospires. (A) L. interrogans containing empty pMaOri.dCas9 (control) or knockdown mutants to LipL32 (pMaOri.dCas9sgRNAlipL32), LipL41 (sgRNAlipL41), and LipL21 (sgRNAlipL21) were incubated with human fibrinogen for binding evaluation by ELISA. Reactivity was revealed with o-phenylenediamine substrate, and the residual component mass in the supernatant was used to infer the mass bound to the leptospiral surface. Statistical analysis was performed using one-way ANOVA, followed by the Tukey post-test for pairwise comparisons of each group against control dCas9, and a P-value of < 0.05 (*) was considered to be statistically significant. (B) Leptospires were resuspended in a human fibrinogen solution (1 mg/mL) to a final OD420nm of 0.5. After 2 h incubation at 37°C, 90 μL of the incubation reactions were added to each well of a microdilution plate and mixed with 10 μL of 0.5 U/mL human thrombin. Experiments were performed with six replicates, and controls with no leptospires (maximal fibrin clot formation) or no thrombin (no fibrin clot formation) were included. Plates were read at 600 nm at 2-min intervals. The results are expressed in percentage of clot formation, where clot turbidity in the control with no leptospires was considered 100%. [file Image_3.JPEG]

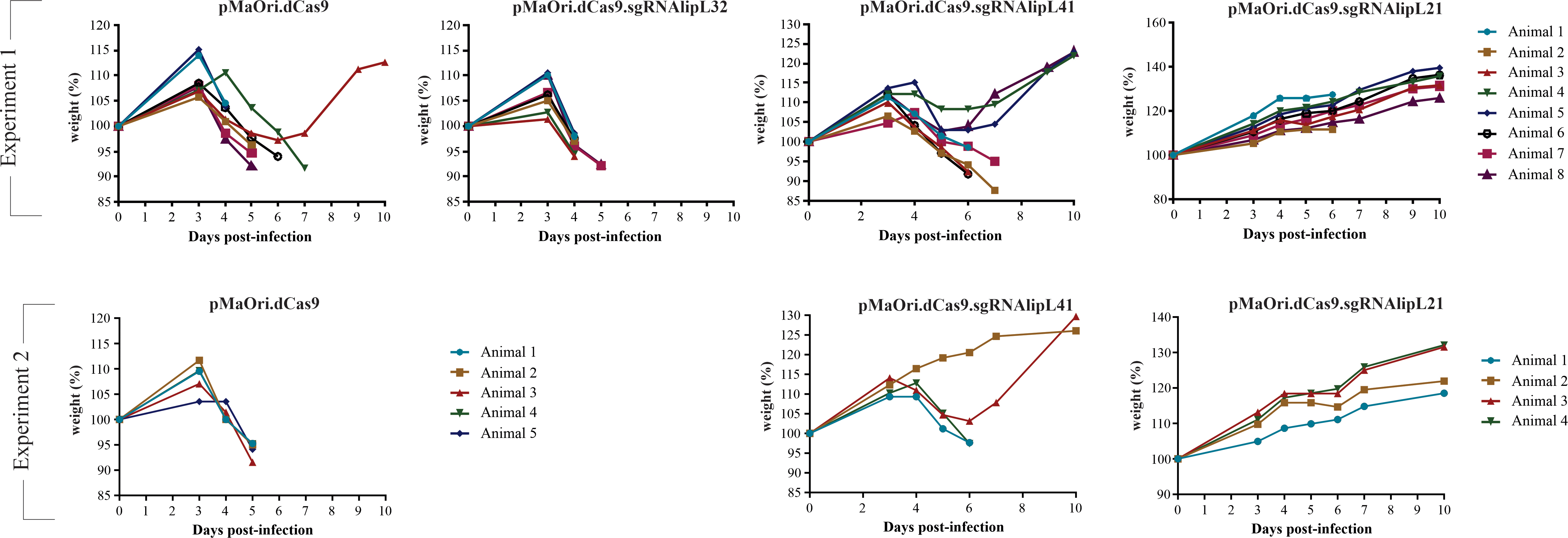

Supplement: Supplementary Figure 4 — Daily measurements of hamsters' weight. Animals' weight from the first and second experiments were recorded daily. For each animal, individual weight displayed at day 0 was considered 100%. LipL32 mutant was not included in the second experiment. [file Image_4.JPEG]

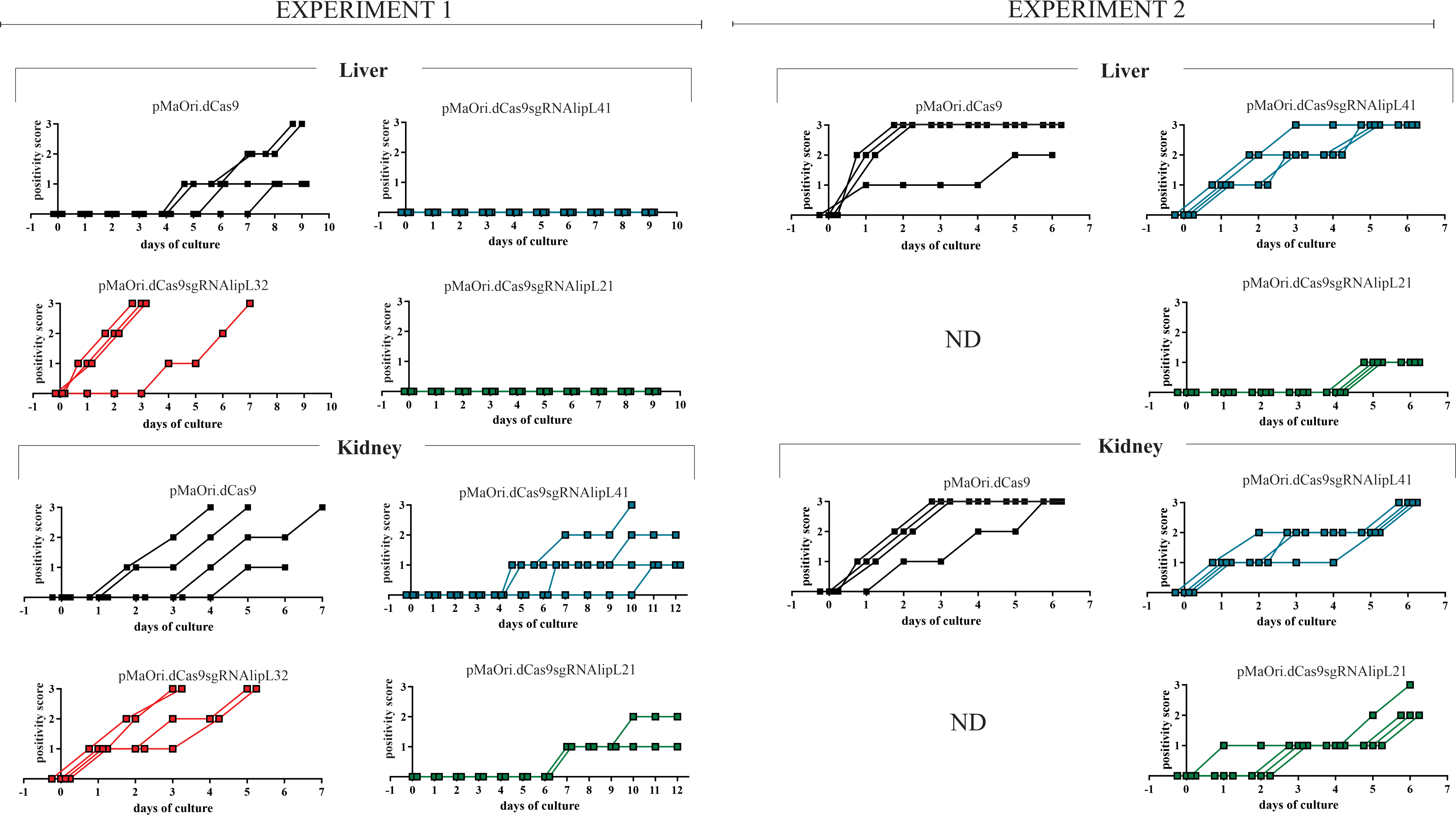

Supplement: Supplementary Figure 5 — Daily observation and positivity scores from medium inoculated with tissue macerates. Cultures containing spectinomycin (retrieval of only mutant leptospires) were monitored daily for the presence and abundance of leptospires, and positivity scores were recorded. 1: 1 to 10 leptospires per field; 2: 10 to 100; 3: >100 leptospires per field. LipL32 mutant was not included in the second experiment. [file Image_5.JPEG]
